# The cultivation conditions of leafy vegetables influence the structures of phyllosphere bacterial communities and ultimately impact the *L. monocytogenes* growth post-harvest

**DOI:** 10.3389/fmicb.2025.1516740

**Published:** 2025-06-30

**Authors:** Paul Culliney, Achim Schmalenberger

**Affiliations:** Department of Biological Sciences, University of Limerick, Limerick, Ireland

**Keywords:** variety, season, lactic acid bacteria, *Pseudomonadaceae*, *Spinacia oleracea*, *Eruca sativa*, *Listeria monocytogenes*

## Abstract

Cultivation conditions, including plant species, variety, cultivation method, and seasonality, are all at least co-factors of epiphytic *Listeria monocytogenes* growth. Meanwhile, phyllosphere-associated bacteria were found to influence the colonization of invading pathogens. Thus, the main objective of this study was to determine whether cultivation conditions are factors in the development of the bacterial phyllosphere community on leafy vegetables, which consequently correlates positively or negatively with *L. monocytogenes* growth. Indeed, this study revealed that vegetable cultivation conditions are a more significant determinant of phyllosphere development than plant species. Of the identified phyllosphere-associated bacteria, the presence of Pseudomonadaceae had a positive correlation with *L. monocytogenes* populations on all tested produce. Hitherto, *Pseudomonadaceae* content appeared to be more critical for *L. monocytogenes* growth on spinach F1 Trumpet. For days 7–9 of storage, *Pseudomonadaceae* increased abundance on open field spinach F1 Trumpet were associated with *L. monocytogenes’* most significant increase (0.94 log_10_ colony-forming unit (cfu) g^−1^). In contrast, *Pseudomonadaceae* content decreased for polytunnel spinach F1 Trumpet, and the corresponding *L. monocytogenes* populations remained unchanged. *Carnobacteriaceae* were present on spinach F1 Trumpet from the polytunnel but not on other spinach products, with higher associated *L. monocytogenes* growth. *Pectobacteriaceae* (genus *Dickeya*) increased for spinach F1 Trumpet polytunnel but decreased for other spinach produce with lower associated *L. monocytogenes* growth. Similarly, polytunnel rocket Esmee had an increasing relative abundance of *Pectobacteriaceae*, whereas it remained constant for polytunnel rocket Buzz. Compared to summer spinach F1 Trumpet produce, winter produce had significantly greater *Streptococcaceae* content and was correlated with a decrease in *L. monocytogenes* growth. Finally, higher phyllosphere alpha diversity putatively limited *L. monocytogenes* growth. Ultimately, this study revealed that cultivation conditions determine the bacterial phyllosphere community structure, which consequently influences *L. monocytogenes* growth.

## 1 Introduction

Leafy vegetables such as rocket and spinach are commonly consumed due to their vitamin, mineral, antioxidant, and phytochemical content (Colonna et al., 2016; Van der Avoort et al., 2018; Venu et al., 2019). To meet the demand for such leafy vegetables, global production of spinach has increased by 218% from 2001 to 2021 (FAO, 2021). Polytunnels enable all year-round production of such high-quality leafy vegetables in winter months or in countries where production may not be possible due to challenging weather conditions (Sagar, 2020). However, cultivation in polytunnels is also altering environmental conditions not only for plant growth but also for the growth of the plant microbiome.

While the increasing demand for vegetables has resulted in the adoption of cost-effective and fast production methods, less concern is given to the safety of their produce, that is, microbial contamination with foodborne pathogens such as *Listeria monocytogenes* (Balali et al., 2020). Potential sources of contamination include irrigation water and manures (re-harvest), as well as the handling of the produce (post-harvest) (Balali et al., 2020). In terms of *L. monocytogenes* growth on spinach and rocket produce, there have been conflicting results from studies with differing experimental and pre-harvest cultivation conditions (Sant'Ana et al., 2012; Lokerse et al., 2016; Söderqvist et al., 2017b; Ziegler et al., 2019; Culliney and Schmalenberger, 2020). However, Culliney and Schmalenberger (2022) revealed that cultivation conditions, that is, plant species and variety, cultivation method (polytunnel vs. open field), and seasonality of harvest, are at least partly responsible for differing levels of *L. monocytogenes* growth (Culliney and Schmalenberger, 2022).

Foodborne pathogens, such as *L. monocytogenes* do not grow in isolation but within a microbial community within the phyllosphere. The phyllosphere refers to the aerial parts of the plant, primarily the surface of the leaves, which harbor diverse and rich communities of bacteria, fungi, viruses, nematodes, and protozoans (Bashir et al., 2022). Plant species and genotype, as well as abiotic factors, such as geographical location, solar radiation, pollution, and nutrients, and biotic factors, including leaf age and presence of other microorganisms, are all drivers of the development of the phyllosphere (Xu et al., 2022). Although the phyllosphere harbors a highly diverse community, at the phylum level, the phyllospheres of different plant species, even from various geographical locations, exhibit high levels of similarity. They primarily consist of *Pseudomonadota* (*Proteobacteria*), *Actinomycetota* (*Actinobacteria*), *Bacteroidota* (*Bacteroidetes*), and *Bacillota* (*Firmicutes*) (Liu et al., 2020).

Phyllosphere-inhabiting microorganisms and their metabolites interact with their environment and may play protective roles against invading opportunistic foodborne pathogens (Saleem, 2021). A previous study revealed that bacterial isolates from ready-to-eat (RTE) lettuce influence the colonization of *Listeria innocua* in co-cultures (Francis and O’Beirne, 2002). However, a paucity of studies has investigated the *in situ* influence of the food microbiome or vegetable phyllosphere on the *L. monocytogenes* growth. A cultivation-based study did not identify any differences in resident bacteria present between cut leaves of broad-leaved endive associated with high and low levels of *L. monocytogenes* growth (Carlin et al., 1995). To date, there have been no attempts to correlate the phyllosphere bacteriome of rocket or kale with *L. monocytogenes* growth.

Lactic acid bacteria (LAB) are often naturally present as indigenous, spoilage bacteria and negatively impact *L. monocytogenes* due to their competitive growth capabilities (Østergaard et al., 2014). Additionally, LAB produce organic acids which reduce pH by lowering intracellular dissociation and intracellular leakage through porins or permeases to values beneath the pH at which *L. monocytogenes* performs optimally, that is, pH 7 (Webb et al., 2022). Moreover, LAB produce other metabolites or bio-preservative agents such as reuterin, bacteriocins, diacetyl, reutericyclin, organic acids, acetoin, and hydrogen peroxide (Ibrahim et al., 2021). *Lactiplantibacillus plantarum* is a LAB previously isolated from rocket produce, which harbors genes that encode for the production of Coagulin A and the active peptide Pediocin ACH. These can act as anti-listerial agents, thus displaying particular inhibition capacities of *L. monocytogenes* (Le Marrec et al., 2000; Espitia et al., 2016; Barbosa et al., 2021). Conversely, several members of the *Pseudomonadaceae* family cause hydrolysis of proteins, which could provide free amino acids likely to stimulate the *L. monocytogenes* growth (Marshall et al., 1992; Zilelidou and Skandamis, 2018). *Pseudomonadaceae* spp. can also increase nutrient availability, for example, carbon and nitrogen for pathogen colonization by altering ion transport across the plant cell plasma membranes (Hutchison, 1995). Additionally, *P. putida* has the ability to produce and release plant growth regulators, for example, indole-3-acetic acid, which promotes nutrient leakage and microbial fitness (Brandl and Lindow, 1998; Leveau and Lindow, 2005). Further research is needed to determine whether a higher diversity of the phyllosphere indigenous bacterial community is related to the reduction of the competitiveness of transient opportunistic pathogenic microorganisms (Darlison et al., 2019).

The objective of the present study was to utilize Illumina-based 16S amplicon sequencing to describe the bacterial composition of leafy vegetable phyllospheres. Different plant species (spinach, rocket, and kale), cultivars (F1 Trumpet vs. F1 Cello; and Buzz vs. Esmee), cultivation methods (polytunnel vs. open field), and seasonality (summer vs. winter spinach) were tested to identify the presence of certain bacteria of importance to *L. monocytogenes* growth. Changes in their relative abundance were correlated with shifts in the abundance of *L. monocytogenes* populations. This study hypothesized that differences in the relative abundance of certain phyllosphere-associated bacterial taxa attributed to differing cultivation conditions are essential co-factors responsible for divergent levels of *L. monocytogenes* growth. Consequently, the present study aimed to analyze the bacterial community structures of leafy vegetables cultivated differently, including spinach, rocket, and kale.

## 2 Materials and methods

### 2.1 Spinach, rocket, and kale produce

All spinach, rocket, and kale produce (Caryophyllales for spinach, Brassicales for rocket, and kale, referred here as species) used in this study were cultivated as described by Culliney and Schmalenberger (2022). A total of 160 samples from *L. monocytogenes* growth potential experiments were selected: open field and polytunnel spinach (F1 Trumpet; summer harvest), open field and polytunnel rocket (Buzz), polytunnel spinach (F1 Cello), polytunnel rocket (Esmee), open field spinach (F1 Trumpet; winter harvest). Samples were stored for days 0, 2, and 5 at 7°C and for days 7–9 at 12°C for days, where *L. monocytogenes* and total bacteria counts (TBCs) were enumerated on cultivation media (Culliney and Schmalenberger, 2022).

### 2.2 *L. monocytogenes* content of spinach, rocket, and kale produce

Growth experiments were executed as described in accordance with the European Union (EU) guidance document’s guidelines for conducting growth potential studies (European Union Reference Laboratory for *Listeria monocytogenes* (EURL Lm); EURL Lm, 2019). The rationale behind selecting these guidelines is to provide a robust representation of real-life scenarios involving low-level contaminations with the potential to grow under realistic storage conditions. Each sample consisted of 25 g of produce inoculated with 100 cfu g^−1^ of a three-strain mix of *L. monocytogenes*, that is, 959 (vegetable isolate), 1,382 (EURL *Lm* reference strain), and 6,179 (food processing plant isolate). The contents of each were transferred into separate stomacher bags and homogenized in 25 mL of phosphate-buffered saline (PBS) using a stomacher (Seward 400, AGB Scientific, Dublin, Ireland) for 120 s at a high speed (260 rpm). These homogenates were used for all types of microbial analysis.

Growth potentials (log_10_ cfu g^−1^) calculated from median values were open field spinach (F1 Trumpet; summer harvest) = 2.59, polytunnel spinach (F1 Trumpet) = 1.40, open field rocket (Buzz) = 1.28, polytunnel rocket (Buzz) = 1.45, polytunnel rocket (Esmee) = 1.23, polytunnel spinach (F1 Cello) = 1.84, polytunnel kale (Nero di Toscana) = 2.56, and open field spinach (F1 Trumpet; winter harvest) = 1.65 as described recently (Culliney and Schmalenberger, 2022).

The associated average *L. monocytogenes* counts (log_10_ cfu g^−1^) across the five time points (± the relative increase or decrease from the previous time point) are displayed in Table 1.

**Table 1:** Average *Listeria monocytogenes* counts (log_10_ cfu g^−1^ ± the relative increase or decrease from the previous time point) over time.

Product	Day 0	Day 2	Day 5	Day 7	Day 9
Open field spinach (F1 Trumpet; summer harvest)	1.99	2.31 (+0.32)	2.90 (+0.59)	3.48 (+0.58)	4.58 (+1.10)
Polytunnel spinach (F1 Trumpet)	1.94	3.04 (+1.10)	3.34 (+0.30)	3.33 (− 0.01)	3.36 (+0.03)
Open field rocket (Buzz)	1.89	2.45 (+0.56)	2.69 (+0.24)	3.14 (+0.45)	3.23 (+0.09)
Polytunnel rocket (Buzz)	1.91	2.48 (+0.57)	2.94 (+0.46)	3.45 (+0.51)	3.52 (+0.07)
Polytunnel rocket (Esmee)	1.94	2.32 (+0.38)	2.78 (+0.46)	2.89 (+0.11)	3.29 (+0.40)
Polytunnel spinach (F1 Cello)	1.91	2.77 (+0.86)	3.30 (+0.53)	3.38 (+0.08)	3.88 (+0.50)
Polytunnel kale (Nero di Toscana)	2.02	2.78 (+0.76)	3.55 (+0.77)	4.03 (+0.48)	4.48 (+0.45)
Open field spinach (F1 Trumpet; winter harvest)	2.12	2.69 (+0.57)	3.24 (+0.55)	3.33 (+0.09)	3.76 (+0.43)

Adapted from Culliney and Schmalenberger (2022).

### 2.3 DNA extraction

The remaining homogenate suspensions obtained after microbial analysis were transferred into 50 mL conical tubes and centrifuged at 4,500*g* (15 min at 4°C). Supernatants were discarded, and the derived pellets were stored at −20°C. For DNA extraction, pellets were resuspended in 400 μL of PBS, and 100 μL was used for DNA extraction with the PowerFood DNA Isolation kit (MO BIO Laboratories, Carlsbad, CA, USA) according to the manufacturer’s instructions. The quantity and quality of the extracted DNA were determined with the Take3 plate in an Eon plate reader/incubator (BioTek, Winooski, VT, USA) (Culliney and Schmalenberger, 2024).

### 2.4 Next generation sequencing (NGS) analysis

All 160 samples were sent to the University of Minnesota Genomics Center (UMGC) for indexing and Illumina MiSeq (San Diego, CA) sequencing. Raw sequencing files were deposited in the Sequence Read Archive (National Center for Biotechnology Information [NCBI]) under the BioProject identification [ID] number: PRJNA117723.4. Bioinformatics analysis was performed using QIIME2 2021.11 (https://qiime2.org/) (Bolyen et al., 2019) as described recently by Culliney and Schmalenberger (2024). The paired-end sequences with quality of each group of 20 samples were demultiplexed and imported with metadata separately via a ManifestPhred33V2 file. This was followed by trimming and truncating (quality filtering at Q20) using the q2-dada2 plugin. Following this, the “qiime feature-table merge” and “qiime feature-table merge-seqs” plug-ins to merge feature tables and the representative amplicon sequence variants (ASV) were conducted, so the following group comparisons could be performed:

Comparison 1 (open field vs. polytunnel vs. plant species) consisted of open field spinach (F1 Trumpet), polytunnel spinach (F1 Trumpet), open field rocket (Buzz), and polytunnel rocket (Buzz).

Comparison 2 (variety vs. species) consisted of polytunnel spinach (F1 Trumpet), polytunnel rocket (Buzz), polytunnel rocket (Esmee), polytunnel spinach (F1 Cello), and polytunnel kale (Nero di Toscana).

Comparison 3 (seasonality) included open field spinach (F1 Trumpet; summer harvest) and open field spinach (F1 Trumpet; winter harvest).

Assigning taxonomic information to the ASV sequences was conducted using a pre-trained Naïve Bayes taxonomic classifier, which was trained on the Silva version 138.99% reference dataset where sequences were trimmed to represent only the region between the 515F/806R primers (V3–V4 region) as described previously (Culliney and Schmalenberger, 2025). Sequences not assigned to a phylum level, chloroplast, and mitochondrial sequences were removed using the filter-table method in the q2-taxa plugin. All subsequent analyses were conducted with both rarefied and unrarefied data. Even sampling depths for use in diversity metrics were for comparison 1: 11,519 → Retained 921,520 (29.48%) features in 80 (100.00%) samples at the specified sampling depth; for comparison 2: 3,117 → Retained 240,009 (9.99%) features in 77 (79.38%) samples at the specified sampling depth; and for comparison 3: 15,015 → Retained 600,600 (40.99%) features in 40 (100.00%) samples at the specified sampling depth. Alpha diversity metrics (observed ASVs, Shannon index, Pielou’s evenness, and Faith’s Phylogenetic Diversity) and beta diversity metrics (weighted unique fraction metric or UniFrac (Lozupone et al., 2007) and Bray–Curtis dissimilarity) using q2-diversity were estimated and viewed on Principal Coordinates Analysis (PCoA) Emperor plots. Analysis of Composition of Microbiomes (ANCOM) test in the q2-composition plugin was used to identify differentially abundant features. ANCOM identified individual taxa whose relative abundances are significantly different across groups. Relative abundance was calculated after conversion of the biome tables from QIIME2 to tsv files (phylum and family levels). Pearson’s correlation coefficient was determined to measure the strength and direction of the linear association between two variables (i.e., between *L. monocytogenes* populations and the corresponding relative abundance of each of the 20 most abundant families) for all groups over time (Sedgwick, 2012). Pearson’s correlation coefficient from <0.10 is a negligible correlation, 0.10–0.39 indicates weak correlations, 0.40–0.69 represents moderate correlations, while 0.70–0.89 indicates strong correlations, with >0.90 being very strong (Schober et al., 2018). Absolute abundances of bacterial taxa at the family and genus level were estimated by using the total heterotrophic counts published elsewhere (Culliney and Schmalenberger, 2022). Input, filtered, denoised, merged, non-chimeric reads, as well as chloroplast to total DNA content, are reported in Supplementary Tables S1–S3.

### 2.5 Statistical analysis

RStudio software (Posit, Boston, MA; version 4.1.1) was used for statistical analysis. In situations of normality (Shapiro–Wilk test) and homoscedasticity (Levene’s), a one-way analysis of variance (ANOVA) was conducted to compare input, filtered, denoised, merged, and non-chimeric reads between groups. The remainder of the statistical analysis for alpha and beta diversity metrics was conducted in QIIME2. For alpha diversity (observed ASVs, Shannon index, Pielou’s evenness, and Faith’s Phylogenetic Diversity (Faith, 1992)), comparisons among groups and pairwise comparisons were conducted through Kruskal–Wallis tests. Beta diversity was analyzed through the non-parametric permutation test, permutational multivariate analysis of variance (PERMANOVA) (999 permutations) (Anderson, 2017). Statistical significance was tested at *p* ≤ 0.05. In situations of normality (Shapiro–Wilk test) and homoscedasticity (Levene’s), a one-way ANOVA Tukey honestly significant difference (HSD) *post hoc* test applying Benjamini–Hochberg correction for multiple testing was conducted to compare relative abundances for all alpha diversity metrics and relative abundances across subgroups. In situations of non-normality, the Kruskal–Wallis rank sum test, using the kruskal.test function, and Dunn test *post hoc* analysis for multiple pairwise comparisons between groups were conducted, applying the Benjamini–Hochberg correction for multiple testing (false discovery rate was set at 10%). In situations of unequal variance, the oneway.test function was employed with var = F, and Games–Howell *post hoc* analysis.

## 3 Results

### 3.1 Comparison 1 (open field vs. polytunnel vs. plant species)

This section describes how alpha and beta diversities were shaped by the selection of different leafy vegetable plants (spinach and rocket) as well as other cultivation methods (open field and polytunnel) and how diversities evolved with storage. The primary assumption was that both plant species and environment affect alpha and beta diversities. In turn, differently developing phyllosphere communities were expected to affect *L. monocytogenes* growth over time, as well as the succession of the phyllosphere community over time.

#### 3.1.1 Influence of cultivation method (polytunnel and open field) and plant species (spinach and rocket) on alpha diversity of a *L. monocytogenes* inoculated phyllosphere

On average, the richness and diversity for rocket (observed features, Shannon index and Faith’s Phylogenetic Diversity) were significantly greater for open field rocket (*L. monocytogenes* growth potential = 1.28 log_10_ cfu g^−1^) compared to polytunnel rocket produce (*L. monocytogenes* growth potential = 1.45 log_10_ cfu g^−1^). Pielou’s evenness was not significantly different between the open field rocket and polytunnel (*p* > 0.05). For spinach, Pielou’s evenness and diversity (Shannon index) were significantly higher for polytunnel spinach (*L. monocytogenes* growth potential = 1.40 log_10_ cfu g^−1^) compared to open field spinach (*L. monocytogenes* growth potential = 2.59 log_10_ cfu g^−1^). However, average observed features and Faith’s Phylogenetic Diversity values did not differ between spinach produce (*p* > 0.05). Except for Pielou’s evenness and Shannon index of polytunnel rocket, no other significant differences over time were observed for all alpha diversity metrics. Significant changes over time were only identified for polytunnel rocket (Shannon index and Pielou’s evenness) and open field rocket (Pielou’s evenness) (Supplementary Table S4), Rarefaction of sequencing reads did not influence alpha diversity metrics (Supplementary Table S5).

#### 3.1.2 Influence of cultivation method (polytunnel and open field) and plant species (spinach and rocket) on beta diversity of a *L. monocytogenes* inoculated phyllosphere

**Figure 1:**
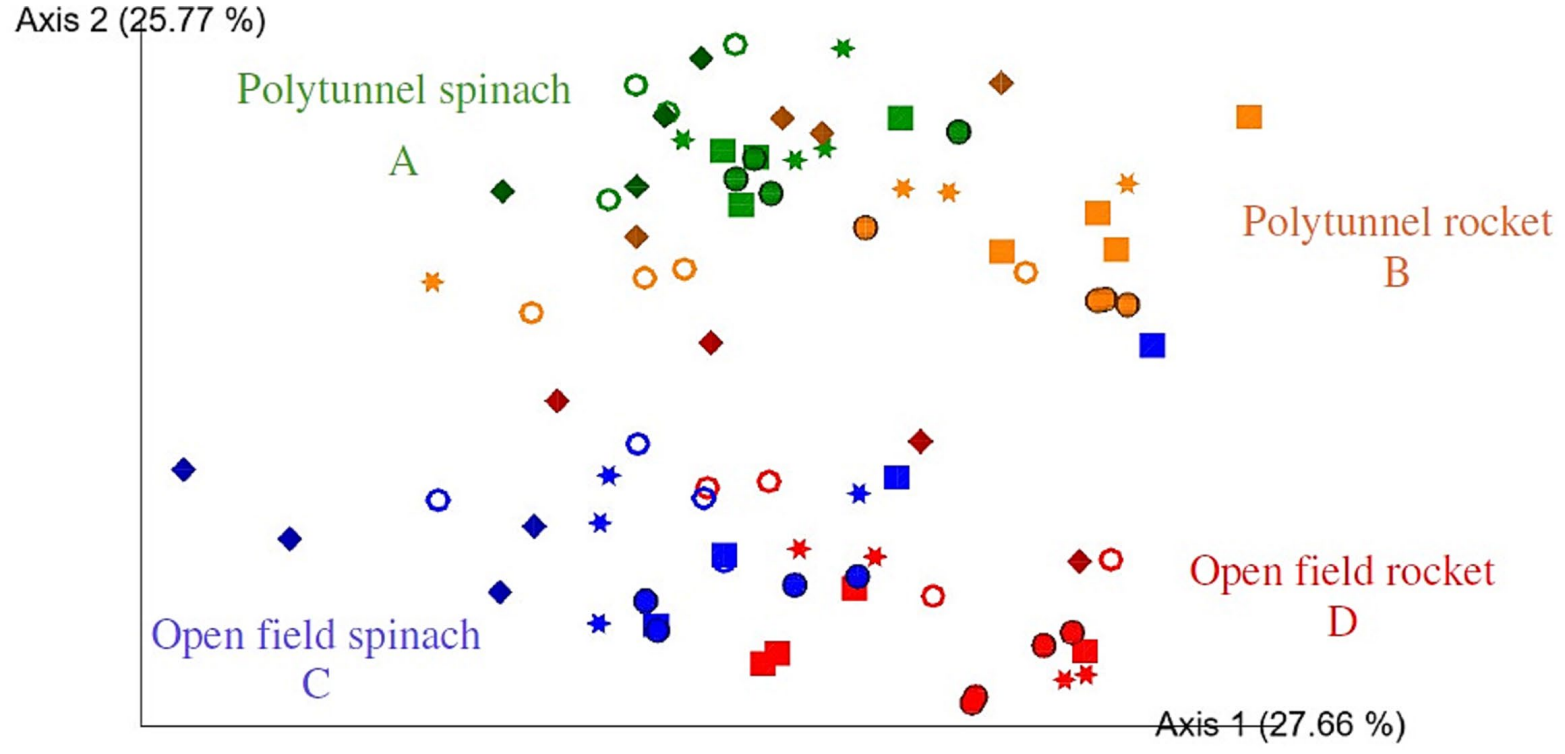
Two-dimensional Emperor (PCoA) plots showing beta diversity distances, that is, weighted UniFrac, among the different samples across open field rocket Buzz (red), open field spinach F1 Trumpet (blue), polytunnel rocket Buzz (orange), and polytunnel spinach F1 Trumpet (green) groups with rarefaction applied. Shapes revealed separations over time are day 0 = circle, day 2 = square, day 5 = star, day 7 = ring, and day 9 = diamond. Letters A-D indicate significant differences.

All four groups, that is, open field rocket, polytunnel rocket, open field spinach, and polytunnel spinach produce, were all significantly different from each other (*p* = 0.001). When grouped by produce type, spinach and rocket produce were also significantly different (*p* = 0.001). Furthermore, when grouped by cultivation method, all polytunnel produce vs. all open field bacterial communities were significantly different (*p* = 0.001). While all four produce groups were significantly different, this was not always the case when compared at individual time points. The bacterial communities of all 5 time points of open field spinach were significantly different compared to polytunnel spinach produce (*p* = 0.026–0.038). The same was observed for polytunnel rocket compared to polytunnel spinach produce (*p* = 0.029–0.035). However, for open field vs. polytunnel rocket, significant differences between their bacterial communities were limited to days 0, 2, 5, and 9 (*p* = 0.019–0.037) and not day 7 (*p* = 0.057). Bacterial communities of open field rocket and spinach showed significant differences on day 0, 7, and 9 (*p* = 0.024–0.030) but not on day 5 or 7 (*p* = 0.069–0.084). Adjusting the *p*-value significance threshold with Benjamini–Hochberg correction did not change the statistical outcome of the tests. A visual representation of the bacterial beta diversity on a PCoA plot showed that communities of polytunnel rocket and spinach, as well as open field spinach and rocket, partially overlapped, while polytunnel rocket and open field rocket, as well as polytunnel spinach and open field spinach, were separated (Figure 1). Overall, beta diversity analyses highlighted the differences between the bacterial phyllosphere communities at the plant species and environment levels.

The phyllosphere of open field rocket produce changed significantly over time, that is, from days 0–9 and 2–9 (*p* = 0.028 and 0.030). For polytunnel rocket produce changes in phyllosphere structure occurred from days 0–9, 2–7, and 2–9 (*p* = 0.021–0.048). Open field spinach produce demonstrated significant changes in its phyllosphere from days 0–9, 2–9, and 5–9 (*p* = 0.014–0.030). Finally, polytunnel spinach exhibited the most significant changes in its phyllosphere community over time, that is, days 0–5, 0–7, 0–9, 2–7, 2–9, 5–7, and 5–9 (*p* = 0.019–0.041). Correcting the *p*-value significance threshold with Benjamini–Hochberg correction changed the outcome of only two statistical tests to non-significant, that is, polytunnel spinach produce from days 0–7 and polytunnel rocket produce from days 7–9. Overall, these findings demonstrate that bacterial phyllosphere communities change substantially during the storage period, even if they do not significantly change at each sampling time.

#### 3.1.3 Influence of cultivation method (polytunnel and open field) and plant species (spinach and rocket) on phyla and family relative abundances of a *L. monocytogenes* inoculated phyllosphere

**Figure 2:**
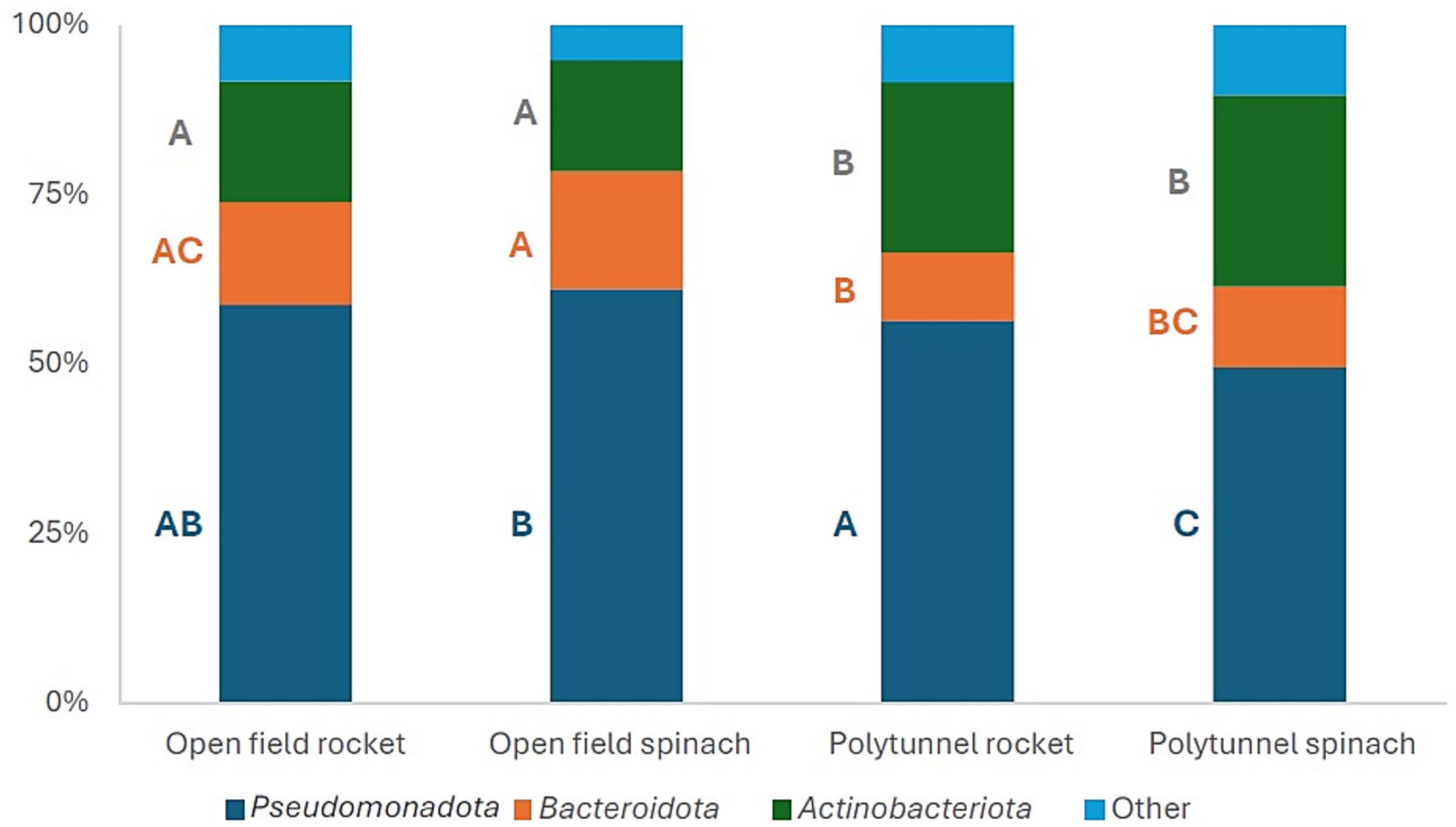
Mean relative abundances (%) of the three most abundant phyla of the 16S gene of the open field rocket Buzz, open field spinach F1 Trumpet, polytunnel rocket Buzz, and polytunnel spinach F1 Trumpet groups, with rarefaction applied. All remaining lower abundant phyla are combined in “Other.” A–C indicate significant differences between groups.

For all four groups, the three most abundant phyla were *Pseudomonadota*, *Actinomycetota*, and *Bacteroidota*, which comprised 89.64–94.82% of the phyllosphere bacterial communities (Figure 2). Over time, the total abundance of these three most abundant phyla ranged for (i) open field rocket from 88.96 to 94.44%, (ii) open field spinach from 92.28 to 96.15%, (iii) polytunnel rocket from 87.71 to 95.02%, and (iv) for polytunnel spinach from 88.21 to 91.26% of total phyla. At the phylum level, cultivation methods appeared to be a more influential determinant of relative bacterial community structure compared to plant species (Figure 2).

A total of 35 families common to all four groups were detected, albeit with some significant differences across groups and low relative abundances (Supplementary Table S6). Out of the 20 most abundant families of each group, 12 were shared by all four groups with substantially higher relative abundances. Open field rocket and polytunnel rocket produce shared 14 of their 20 most abundant families, four of which were significantly different in relative abundance. Open field rocket and open field spinach produce shared 16 families of their 20 most abundant, eight of which had significantly different relative abundances between the two groups. Open field spinach and polytunnel spinach produce had 16 families of their most abundant 20 in common, nine of which were significantly different. Polytunnel rocket and polytunnel spinach shared 16 out of 20 most abundant families, seven of which were significantly different (Table 2). Of the 15 families that showed differences in relative abundance, eight appeared to group by cultivation type (polytunnel and open field), while only four grouped by plant species. However, when total heterotrophic counts were used to estimate total abundances, the higher total abundance of bacteria in the spinach phyllospheres (open field and polytunnel) resulted in all but one family grouping according to plant species (Table 3 and Supplementary Table S7).

**Table 2:** Average relative abundance ± the standard error of families present different relative abundances in the phyllosphere of the open field vs. polytunnel and rocket vs. spinach with rarefaction applied. Letters a-c indicate significant differences.

Family	Rocket	Spinach	Rocket	Spinach
	Open fields	Open fields	Polytunnel	Polytunnel
*Hymenobacteraceae*	6.08 ± 0.67^a^	5.05 ± 0.76^a^	1.93 ± 0.32^b^	0.48 ± 0.09^c^
*Rhizobiaceae*	4.53 ± 0.39^a^	2.96 ± 0.24^b^	4.07 ± 0.39^ab^	3.76 ± 0.32^ab^
*Sphingobacteriaceae*	3.78 ± 0.37^ab^	6.26 ± 0.93^b^	2.39 ± 0.47^a^	5.82 ± 0.60^b^
*Microbacteriaceae*	5.48 ± 0.33^a^	8.01 ± 0.51^b^	5.37 ± 0.41^a^	9.83 ± 0.60^c^
*Pectobacteriaceae*	3.28 ± 0.29^a^	7.79 ± 1.16^b^	2.20 ± 0.21^a^	7.46 ± 1.05^b^
*Caulobacteraceae*	0.54 ± 0.05^a^	0.81 ± 0.10^a^	2.79 ± 0.25^b^	4.51 ± 0.30^c^
*Xanthomonadaceae*	1.04 ± 0.14^a^	2.65 ± 0.28^b^	1.61 ± 0.37^a^	1.39 ± 0.13^a^
*Nocardiaceae*	5.85 ± 0.46^a^	5.76 ± 1.00^a^	8.26 ± 0.74^b^	11.11 ± 0.53^c^
*Sphingomonadaceae*	14.32 ± 0.65^a^	16.63 ± 0.98^a^	8.77 ± 1.15^b^	6.63 ± 0.48^b^
*Comamonadaceae*	2.75 ± 0.33^a^	1.47 ± 0.13^c^	0.96 ± 0.19^b^	0.83 ± 0.13^b^
*Beijerinckiaceae*	12.89 ± 0.66^a^	7.67 ± 1.05^b^	11.84 ± 0.91^a^	3.87 ± 0.45^c^
*Micrococcaceae*	0.78 ± 0.12^a^	0.45 ± 0.05^a^	3.95 ± 0.56^b^	1.47 ± 0.12^c^
*Nocardioidaceae*	1.85 ± 0.18^a^	0.72 ± 0.10^b^	5.71 ± 0.62^c^	2.90 ± 0.14^c^
*Oxalobacteraceae*	1.90 ± 0.22^a^	2.94 ± 0.31^b^	1.01 ± 0.15^c^	1.33 ± 0.17^ac^
*Exiguobacteraceae*	0.52 ± 0.11^a^	0.94 ± 0.28^a^	3.26 ± 0.58^b^	4.68 ± 0.46^b^

Families listed are present with greater than 2% relative abundance in at least one of the four groups.

**Table 3:** Average absolute abundance (log 10, sequence data linked to total cfu counts) of the 20 most abundant families with significantly different abundances in the phyllosphere of the open field vs. polytunnel and rocket vs. spinach with rarefaction applied. Letters a-c indicate significant differences.

Family	Rocket	Spinach
	Open fields	Polytunnel	Open fields	Polytunnel
*Pseudomonadaceae*	6.42^a^	5.97^a^	7.08^b^	7.12^b^
*Sphingomonadaceae*	6.10^ab^	5.44^a^	7.12^c^	6.80^bc^
*Nocardiaceae*	5.83^ab^	5.70^a^	6.70^bc^	7.04^c^
*Pectobacteriaceae*	5.60^a^	5.10^a^	6.76^b^	7.01^b^
*Microbacteriaceae*	5.77^a^	5.28^a^	6.80^b^	6.97^b^
*Sphingobacteriaceae*	5.54^a^	5.09^a^	6.81^b^	6.78^b^
*Beijerinckiaceae*	6.05ab	5.68^a^	6.77^c^	6.51^bc^
*Weeksellaceae*	5.37^a^	5.10^a^	6.60^b^	6.49^b^
*Rhizobiaceae*	5.78^a^	5.43^a^	6.43^b^	6.54^b^
*Paenibacillaceae*	4.40^a^	4.17^a^	6.39^b^	6.55^b^
*Caulobacteraceae*	4.55^a^	4.97^ab^	5.86^bc^	6.64^c^
*Exiguobacteraceae*	4.53^a^	5.07^ab^	5.85^bc^	6.61^c^
*Xanthomonadaceae*	5.00^a^	4.88^a^	6.38^b^	6.20^b^
*Nocardioidaceae*	5.28^a^	5.33^ab^	5.81^bc^	6.48^c^
*Hymenobacteraceae*	5.59^ab^	4.58^a^	6.51^c^	5.57^bc^
*Oxalobacteraceae*	5.15^ab^	4.41^a^	6.41^c^	5.98^bc^
*Flavobacteriaceae*	5.21^a^	4.92^a^	6.30^b^	5.99^b^
*Micrococcaceae*	5.13^a^	5.48^a^	5.58^ab^	6.18^b^
*Comamonadaceae*	5.26^ab^	4.78^a^	6.11^c^	5.85^bc^
*Moraxellaceae*	4.28^a^	5.72^ab^	5.21^ab^	6.02^b^

Overall, *L. monocytogenes* populations for all four groups showed common negative correlations with families *Sphingomonadaceae* and *Beijerinckiaceae* (Table 4 and Supplementary Tables S8–S11). Similarly, only two common positive correlations were identified, namely, *Pseudomonadaceae* and *Xanthomonadaceae*, between all four groups*. L. monocytogenes* populations of open field rocket had a strong positive correlation with three families, whereas a strong to very strong negative correlation was identified with seven families. *L. monocytogenes* populations of polytunnel rocket had a strong positive correlation with five families, and a strong to very strong negative correlation was revealed with seven families. For open field spinach *L. monocytogenes* populations showed a strong to very strong correlation with six families, while strong to very strong negative correlations were identified with only three. Finally, *L. monocytogenes* populations in polytunnel spinach showed a strong positive correlation with four families, and a strong to very strong negative correlation was identified among six families (Table 4).

**Table 4:** Average relative abundance (% ± standard error) of families (16S ribosomal DNA [rDNA]) of open field, polytunnel, spinach and rocket across days 0, 2, 5, 7, and 9 rarefied with strong or very strong Pearson’s correlation coefficient (i.e., the strength and direction of the relationship between that specific family’s relative abundances and the corresponding *Listeria monocytogenes* populations over time). Letters a-c indicate significant differences between the groups.

	Day 0	Day 2	Day 5	Day 7	Day 9	Pearson’s correlation
Open field rocket						
*Sphingomonadaceae*	16.94 ± 0.93^a^	14.43 ± 1.26^ab^	16.01 ± 0.71^a^	13.06 ± 0.87^ab^	11.14 ± 1.59^b^	−0.87, strong
*Beijerinckiaceae*	14.41 ± 0.28^a^	13.25 ± 1.60^a^	15.03 ± 0.95^a^	12.19 ± 1.08^a^	9.56 ± 1.76^a^	−0.73, strong
*Pseudomonadaceae*	5.04 ± 0.57^a^	6.01 ± 1.84^a^	10.45 ± 2.58^a^	13.35 ± 2.99^a^	29.11 ± 6.81^a^	+0.80, strong
*Nocardiaceae*	4.24 ± 0.59^a^	5.36 ± 1.23^a^	5.57 ± 0.54^a^	8.16 ± 0.35^a^	5.93 ± 1.27^a^	+0.79, strong
*Rhizobiaceae*	3.94 ± 0.45^a^	3.47 ± 0.84^a^	3.23 ± 0.70^a^	6.04 ± 0.91^a^	5.95 ± 0.37^a^	+0.73, strong
*Hymenobacteraceae*	9.06 ± 0.80^a^	6.59 ± 1.39^ab^	6.77 ± 1.37^ab^	5.16 ± 0.66^ab^	2.84 ± 1.47^b^	−0.93, very strong
*Comamonadaceae*	3.55 ± 0.79^a^	4.16 ± 0.89^a^	2.42 ± 0.46^a^	2.25 ± 0.37^a^	1.35 ± 0.25^a^	−0.81, strong
*Oxalobacteraceae*	2.64 ± 0.14^a^	1.78 ± 0.27^a^	2.05 ± 0.70^a^	1.99 ± 0.57^a^	1.05 ± 0.37^a^	−0.78, strong
*Chthoniobacteraceae*	2.17 ± 0.40^a^	1.06 ± 0.26^a^	1.68 ± 0.64^a^	1.35 ± 0.64^a^	0.86 ± 0.47^a^	−0.75, strong
*Xanthobacteraceae*	1.19 ± 0.25^a^	0.76 ± 0.12^a^	0.93 ± 0.36^a^	0.61 ± 0.27^a^	0.43 ± 0.16^a^	−0.92, very strong
Polytunnel rocket						
*Sphingomonadaceae*	16.03 ± 2.85^a^	10.47 ± 1.66^ab^	6.75 ± 0.98^b^	5.63 ± 1.33^b^	4.96 ± 0.13^b^	−0.97, very strong
*Beijerinckiaceae*	14.83 ± 1.09^a^	14.69 ± 0.68^a^	10.73 ± 2.37^a^	10.44 ± 1.87^a^	8.49 ± 2.22^a^	−0.93, very strong
*Pseudomonadaceae*	4.46 ± 2.49^a^	4.12 ± 1.73^a^	10.57 ± 1.76^ab^	14.12 ± 2.67^bc^	20.91 ± 3.54^c^	+0.78, strong
*Microbacteriaceae*	7.82 ± 0.91^a^	6.24 ± 0.36^ab^	4.97 ± 0.67^ab^	4.22 ± 0.44^b^	3.62 ± 0.17^b^	−0.99, very strong
*Rhizobiaceae*	3.09 ± 0.15^a^	2.87 ± 0.45^a^	3.68 ± 0.65^a^	4.85 ± 0.80^a^	5.84 ± 1.22^a^	+0.88, strong
*Sphingobacteriaceae*	1.32 ± 0.14^a^	0.77 ± 0.24^a^	2.99 ± 1.30^a^	4.66 ± 1.03^a^	2.22 ± 1.03^a^	+0.70, strong
*Pectobacteriaceae*	1.91 ± 0.61^a^	1.81 ± 0.42^a^	1.95 ± 0.32^a^	2.60 ± 0.57^a^	2.75 ± 0.41^a^	+0.85, strong
*Nocardioidaceae*	6.24 ± 0.78^a^	8.49 ± 1.77^a^	5.58 ± 1.06^a^	4.86 ± 0.87^a^	3.40 ± 1.35^a^	−0.71, strong
*Hymenobacteraceae*	2.61 ± 0.57^ab^	3.37 ± 0.54^a^	1.91 ± 0.48^ab^	1.47 ± 0.81^ab^	0.31 ± 0.12^b^	−0.81, strong
*Moraxellaceae*	4.39 ± 1.49^a^	2.42 ± 0.64^a^	4.79 ± 2.49^a^	7.27 ± 1.48^a^	11.73 ± 2.25^a^	+0.75, strong
*Caulobacteraceae*	3.93 ± 0.36^a^	2.86 ± 0.77^ab^	2.73 ± 0.35^ab^	2.83 ± 0.41^ab^	1.57 ± 0.20^b^	−0.84, strong
*Rhodobacteraceae*	2.91 ± 0.19^ab^	4.21 ± 0.61^a^	2.80 ± 0.47^ab^	1.47 ± 0.23^b^	1.41 ± 0.16^b^	−0.74, strong
Open field spinach						
*Sphingomonadaceae*	19.87 ± 1.27^a^	17.07 ± 2.08^a^	16.97 ± 2.06^a^	16.57 ± 2.71^a^	12.67 ± 2.08^a^	−0.93, very strong
*Beijerinckiaceae*	7.64 ± 1.03^a^	12.12 ± 3.48^a^	8.97 ± 2.37^a^	6.47 ± 1.18^a^	3.14 ± 0.54^a^	−0.81, strong
*Sphingobacteriaceae*	2.96 ± 1.21^a^	3.73 ± 1.57^a^	5.78 ± 1.72^a^	7.04 ± 1.66^ab^	11.80 ± 1.31^b^	+0.99, very strong
*Weeksellaceae*	2.88 ± 1.11^a^	3.99 ± 1.66^a^	3.85 ± 0.91^a^	3.72 ± 0.81^a^	6.11 ± 0.63^a^	+0.88, strong
*Xanthomonadaceae*	2.04 ± 0.54^a^	2.60 ± 0.98^a^	2.20 ± 0.58^a^	3.04 ± 0.46^a^	3.37 ± 0.57^a^	+0.88, strong
Unknown family	1.10 ± 0.36^a^	1.74 ± 0.57^a^	2.86 ± 0.55^a^	5.39 ± 2.73^a^	5.68 ± 2.05^a^	+0.94, very strong
(*Enterobacterales* order)						
*Hymenobacteraceae*	10.15 ± 1.88^a^	5.61 ± 0.38^a^	4.22 ± 0.66^a^	3.13 ± 0.66^a^	2.11 ± 0.84^a^	−0.86, strong
*Flavobacteriaceae*	0.08 ± 0.03^a^	0.22 ± 0.07^ab^	1.34 ± 0.26^ab^	1.74 ± 1.53^ab^	4.52 ± 2.03^b^	+0.97, very strong
*Oxalobacteraceae*	2.69 ± 0.51^a^	2.18 ± 0.66^a^	2.89 ± 0.60^a^	2.61 ± 0.70^a^	4.34 ± 0.82^a^	+0.84, strong
Polytunnel spinach						
*Sphingomonadaceae*	7.74 ± 0.61^ab^	7.20 ± 0.79^ab^	8.59 ± 0.91^a^	5.65 ± 0.71^bc^	3.98 ± 0.77^c^	−0.63, moderate
*Beijerinckiaceae*	6.52 ± 1.43^a^	4.21 ± 0.15^ab^	3.33 ± 0.63^b^	2.76 ± 0.66^b^	2.50 ± 0.24^b^	−0.98, very strong
*Pseudomonadaceae*	4.19 ± 0.56^a^	5.09 ± 0.71^a^	10.31 ± 1.88^b^	15.72 ± 1.93^c^	12.67 ± 1.23^bc^	+0.83, strong
*Microbacteriaceae*	11.41 ± 0.66^a^	10.50 ± 2.03^a^	10.68 ± 1.33^a^	7.93 ± 0.68^a^	8.64 ± 1.29^a^	−0.77, strong
*Pectobacteriaceae*	3.88 ± 1.25^ab^	2.97 ± 1.03^a^	8.43 ± 1.44^ab^	8.73 ± 1.12^ab^	13.29 ± 2.39^b^	+0.87, strong
*Weeksellaceae*	5.01 ± 0.72^a^	4.05 ± 0.72^a^	3.14 ± 0.50^a^	3.07 ± 0.91^a^	2.31 ± 0.47^a^	−1.00, very strong
Unknown family	0.52 ± 0.24^a^	0.57 ± 0.15^a^	2.44 ± 0.77^a^	6.76 ± 2.40^a^	8.08 ± 2.89^a^	+0.83, strong
(*Enterobacterales* order)						
*Oxalobacteraceae*	2.37 ± 0.20^a^	1.80 ± 0.20^a^	1.16 ± 0.08^b^	0.69 ± 0.15^b^	0.69 ± 0.15^b^	−0.96, very strong
*Exiguobacteraceae*	6.90 ± 0.86^a^	4.91 ± 1.11^ab^	5.43 ± 0.35^a^	4.06 ± 0.72^ab^	2.11 ± 0.15^b^	−0.90, very strong
*Rhodobacteraceae*	3.46 ± 0.66^a^	3.06 ± 0.23^a^	2.76 ± 0.24^ab^	1.63 ± 0.15^b^	1.56 ± 0.22^ab^	−0.88, strong
*Sanguibacteraceae*	0.97 ± 0.09^a^	0.94 ± 0.11^a^	1.62 ± 0.19^ab^	1.49 ± 0.22^ab^	2.01 ± 0.31^b^	+0.89, strong

*Pseudomonadaceae* content was not significantly different between all four groups (*p* = 0.277–0.849). On average, open field spinach displayed the highest average *Pseudomonadaceae* content, that is, 13.42%, followed by open field rocket 12.79%, polytunnel rocket 10.44%, and, finally, polytunnel spinach 9.60% (Supplementary Tables S8–S11). Therefore, open field spinach, which displayed the highest growth potential of 2.59 log_10_ cfu g^−1^ was associated with the highest average *Pseudomonadaceae* content, compared to spinach grown in a polytunnel setting, which displayed only 1.40 log_10_ cfu g^−1^. Relative abundance of *Pseudomonadaceae* content was compared for all four groups across the five different time points: At day 0, open fields spinach and open field rocket were significantly different (*p* < 0.001) and open field spinach and polytunnel spinach were significantly different (*p* < 0.001), remaining comparisons were not significantly different (*p* = 0.154–0.995). However, at days 2, 5, 7, and 9, no groups were significantly different from one another (*p* = 0.448–0.896, 0.161–0.984, 0.999, and 0.252–0.748). From days 7–9, *Pseudomonadaceae* content increased for open field rocket from 13.35 to 29.11% and polytunnel rocket from 14.12 to 20.91% (Supplementary Tables S8–S11). Open field spinach showed a moderate correlation (+0.66) between *L. monocytogenes and Pseudomonadaceae* compared to the strong positive correlation in polytunnel spinach. Similarly, open field and polytunnel rocket were strongly positively correlated between the two taxa (Table 4 and Supplementary Tables S8–S11).

Relative *Pectobacteriaceae* content (of which genus *Dickeya* was the sole genus) of polytunnel spinach produce displayed an increasing trend in relative abundance (3.9–13.3%) and strong positive correlation with *L. monocytogenes* populations from days 0–9 compared to open field spinach produce which displayed a decreasing trend (12.6–5.6%) and a moderate negative correlation with *L. monocytogenes* for the same period. The *Pectobacteriaceae* content remained consistently lower for rocket than for spinach. Moreover, the content of *Pectobacteriaceae* was positively correlated with *L. monocytogenes* in polytunnel rocket, which had a higher *L. monocytogenes* growth potential than open field rocket. Indeed, *Pectobacteriaceae* content of open field rocket correlated moderately negatively with decreasing *L. monocytogenes* populations (4.4–4.1%, Table 4 and Supplementary Tables S7–S10).

Polytunnel spinach retained the largest relative content of *Lactobacillales* (order level) (0.31%), followed by open field spinach (0.20%), open field rocket (0.16%), and, finally, polytunnel rocket (0.04%). Only the *Lactobacillales* content of open fields rocket vs. polytunnel rocket and polytunnel spinach vs. polytunnel rocket were significantly different (*p* = 0.019 and 0.027). Moreover, over time, significant differences were observed only at day 2, where the following comparisons were significantly different (*p* = 0.007, 0.019, and 0.019): open field rocket and polytunnel rocket; open field rocket and polytunnel spinach; and polytunnel rocket and open field spinach are significantly different. The relative abundance of *Carnobacteriaceae* (family of *Lactobacillales*) was significantly different between open field rocket and polytunnel spinach, as well as open field spinach and polytunnel spinach (*p* = 0.037 and 0.001), while all remaining group comparisons were not significantly different (*p* = 0.101–0.582). The *Carnobacteriaceae* content was on average 0.26% for polytunnel spinach, but was not at all present in open field spinach. On polytunnel and open field rocket, the relative abundance of *Carnobacteriaceae* was on average 0.01 and 0.02%, respectively.

Although detected and enumerated on *Listeria* selective agar, the *Listeria* genus, belonging to the *Lactobacillales* order, was not detected using NGS on open field spinach or open field rocket produce and was detected on only two of 20 samples belonging to polytunnel rocket produce, and in only one of 20 samples belonging to polytunnel spinach produce.

Overall, the majority of bacterial phyla and families were detected on spinach and rocket in both open fields and polytunnels. However, specific taxa and their change in abundance over time could be correlated with *L. monocytogenes* growth.

### 3.2 Comparison 2 (variety vs. species)

This section describes how their plant hosts shaped alpha and beta diversities at different taxonomic levels, that is, order (Caryophyllales for spinach, Brassicales for rocket and kale, referred here as species) vs. variety (Trumpet and Cello for spinach, Buzz and Esmee for rocket) and how diversity evolved during storage. The primary assumption was that both plant species and variety affect alpha and beta diversities. In turn, differently developing phyllosphere communities were expected to affect *L. monocytogenes* growth over time, as well as the succession of the phyllosphere community over time.

#### 3.2.1 Influence of spinach and rocket cultivars as well as kale on alpha diversity of a *L. monocytogenes* inoculated phyllosphere

Rocket Buzz demonstrated the highest richness and diversity, followed by spinach F1 Trumpet, rocket Esmee, spinach F1 Cello, and, finally, kale Nero di Toscana (Supplementary Table S12). On average, observed features were all significantly different, with kale being the lowest, while rocket Buzz was the highest at day 5. For Faith’s Phylogenetic Diversity, kale Nero di Toscana and spinach F1 Cello were statistically similar; and spinach F1 Trumpet and rocket Esmee were statistically identical (*p* > 0.05). Only spinach F1 Trumpet and rocket Buzz had a significantly higher Shannon index than the remaining leafy vegetables. Spinach F1 Trumpet and rocket Buzz displayed the highest evenness (Pielou’s) that was substantially higher than for rocket Esmee, spinach F1 Cello, and kale Nero di Toscana.

Indeed, kale Nero di Toscana, with the lowest diversity, was associated with increased *L. monocytogenes* (growth potential = 2.56 log_10_ cfu g^−1^), and spinach F1 Cello, with the second-lowest diversity measurements, was associated with the second-highest growth potential, that is, 1.84 log_10_ cfu g^−1^. In contrast, the higher diversity groups spinach F1 Trumpet, rocket Esmee, and rocket Buzz were associated with lower growth potentials of *L. monocytogenes* (i.e., 1.23–1.45 log_10_ cfu g^−1^). Few significant differences were observed over time for alpha diversity metrics, which were limited to rocket Buzz for Shannon diversity (significantly highest on days 5 and 7) and Pielou’s evenness (significantly highest at day 5) (Supplementary Table S12).

The primary observation in this section is that differences in bacterial alpha diversity are related to plant taxonomic relatedness. These, in turn, may limit the growth and potential of *L. monocytogenes* when diversity is high.

#### 3.2.2 Influence of spinach and rocket cultivars and kale on beta diversity of a *L. monocytogenes* inoculated phyllosphere

**Figure 3:**
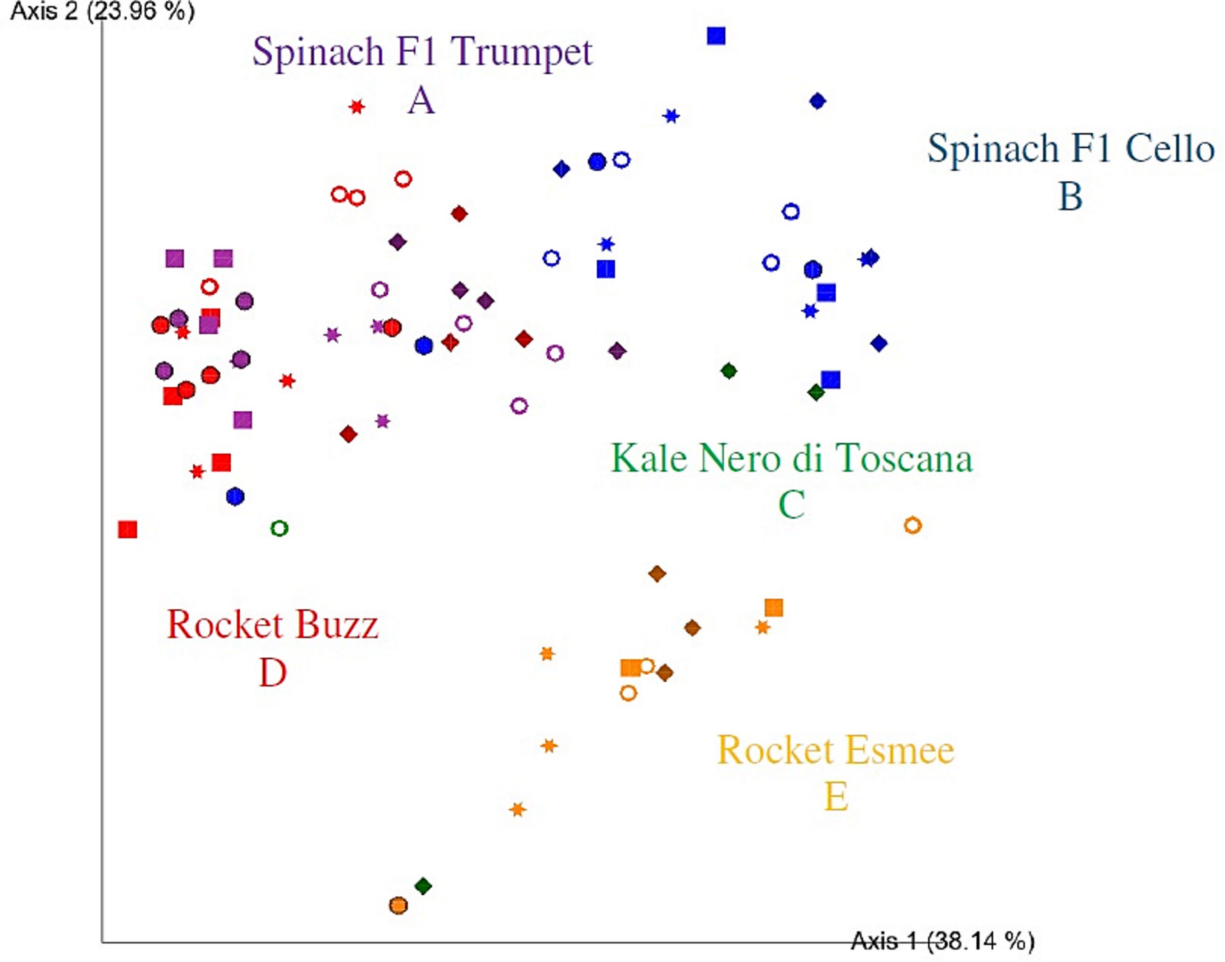
Two-dimensional Emperor (PCoA) plots showing beta diversity distances, that is, weighted UniFrac, among the different samples across polytunnel produce: rocket Esmee (orange), spinach F1 Cello (blue), kale Nero di Toscana (green), rocket Buzz (red) and spinach F1 Trumpet (purple) with rarefaction applied. Shapes revealed separations over time where day 0 = circle, day 2 = square, day 5 = star, day 7 = ring, and day 9 = diamond. 16 and 7 samples with low bacterial reads were removed for kale Nero di Toscana and rocket Esmee, respectively. Letters (A-E) indicate significant differences.

Based on the PCoA bi-plot, rocket Esmee was more separated on axis two from all other groups (Figure 3). Moreover, spinach F1 Cello and spinach F1 Trumpet partially overlapped, while spinach Trumpet also partially overlapped with rocket Buzz. However, significant differences were identified among all bacterial communities (*p* = 0.002–0.036), indicating that bacterial community structures are determined down to the plant variety level. When rocket and spinach varieties were grouped together, respectively, kale and rocket as well as kale and spinach were no longer significantly different (*p* = 0.140 and 0.059, respectively). However, spinach and rocket remained significantly different (*p* = 0.003). Adjusting the *p*-value significance threshold with Benjamini–Hochberg correction did not influence the outcome of the PERMANOVA tests. Comparisons within a vegetable variety over time were compromised for kale Nero di Toscana and rocket Esmee due to low sequence reads on days one to seven and day one, respectively.

#### 3.2.3 Influence of spinach and rocket cultivars and kale on phyla and family relative abundances of a *L. monocytogenes* inoculated phyllosphere

**Figure 4:**
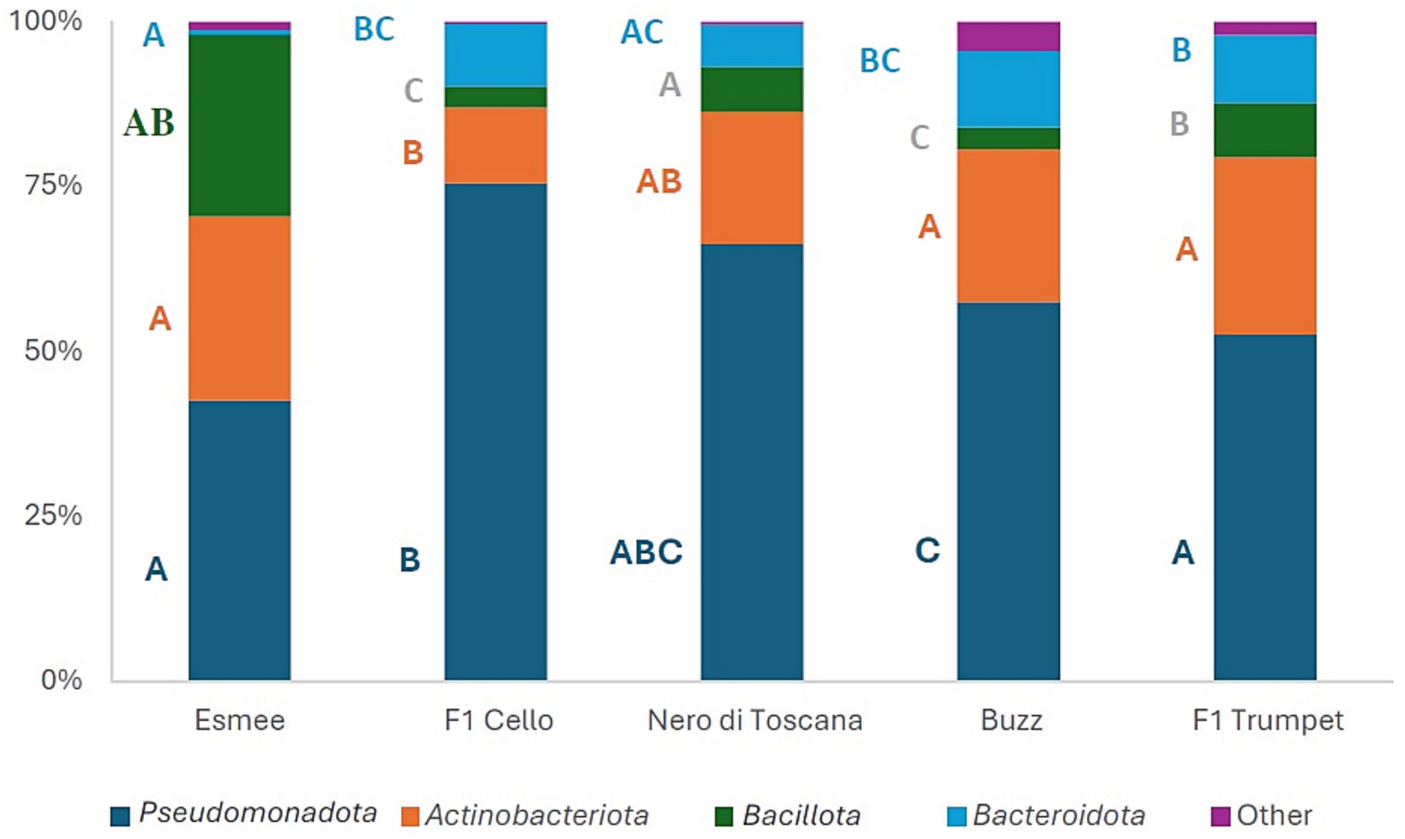
Mean relative abundances (%) of the four most abundant phyla of the 16S gene of the polytunnel produce: rocket Esmee, spinach F1 Cello, kale Nero di Toscana, rocket Buzz, and spinach F1 Trumpet, with rarefaction applied. All remaining lower abundant phyla are combined in “Other,” Letters A to C indicate significant differences between groups.

For all five groups, the four most abundant phyla were *Pseudomonadota*, *Actinobacteriota*, *Bacteroidota*, and *Bacillota*, which comprised 95.61–99.58% of the phyllosphere bacterial communities (Figure 4). Over time, the total abundance of these four most abundant phyla remained consistent across all five groups, ranging from 93.15 to 99.92%.

At the family level, 32 were common to all 5 groups, and their relative abundance was overall significantly affected by the leafy vegetable (Supplementary Table S13). Of the 20 most abundant families, 11, 3, 8, and 0 families showed significant changes in relative abundance over time for spinach F1 Trumpet, spinach F1 Cello, rocket Buzz, and rocket Esmee, respectively (Supplementary Tables S14–S17).

Spinach F1 Cello and Trumpet shared 17 out of the 20 most abundant families, whereas rocket varieties Esmee and Buzz only shared 14 families, of which the relative abundances of 10 were significantly different (*p* < 0.05). Since only four (days 7 and 9) kale samples were obtained with a sufficient number of reads for analysis, comparisons at the family level for kale were avoided.

Overall, *L. monocytogenes* populations for both spinach and rocket varieties exhibited only one common negative correlation with the *Sphingomonadaceae* and one common positive correlation with the *Pseudomonadaceae* (Table 4 and Supplementary Tables S14–S17)*. L. monocytogenes* populations in spinach F1 Trumpet showed a strong positive correlation with *Pseudomonadaceae*, while a strong to very strong negative correlation was identified with six other families (Table 4). *L. monocytogenes* populations of spinach F1 Cello had a strong and very strong positive correlation with *Flavobacteriaceae* and *Pseudomonadaceae*, respectively, whereas a strong negative correlation was identified with four families. *L. monocytogenes* populations of rocket Buzz had a strong positive correlation with five families and a strong to very strong negative correlation with seven families. For rocket Esmee, a strong positive correlation with families *Pseudomonadaceae* and *Xanthomonadaceae* and a strong to very strong negative correlation was observed with eight families (Table 4 and Supplementary Tables S14–S17).

Spinach F1 Cello had an average higher, although not significant, *Pseudomonadaceae* content (19.0%) compared to spinach F1 Trumpet (9.6%). Rocket Esmee had a significantly (*p* < 0.05) higher average *Pseudomonadaceae* content (28.1%) compared to rocket Buzz (10.8%). However, at the genus level, absolute numbers (based on total heterotrophic counts) of *Pseudomonas* sp. are only clearly higher at days 2, 5, and 7 in variety Esmee when compared to Buzz (Supplementary Table S18). For both spinach varieties and rocket Buzz, *Pseudomonadaceae* content appeared to drastically and significantly increase (3.3–5.5-fold) over time. *Pectobacteriaceae* content (genus *Dickeya*) of polytunnel spinach F1 Trumpet produce displayed an increasing trend in relative abundance from days 0–9 (3.8–13.6%) compared to spinach F1 Cello produce, which displayed a decreasing trend (28.5–6.4%) for the same period. Moreover, the *Pectobacteriaceae* content (genus *Dickeya*) of polytunnel rocket Buzz from days 0–9 remained consistent (1.9–2.6%), whereas it increased substantially on rocket Esmee from 1.0 to 6.9%. Spinach F1 Cello had a *Lactobacillales* (order level) content of 0.03% compared to 0.35% for spinach F1 Trumpet. The *Lactobacillales* content of rocket Esmee and rocket Buzz was similar to F1 Cello (0.01 and 0.06%). The average *Carnobacteriaceae* content of spinach F1 Cello (*L. monocytogenes* growth potential = 1.84 log_10_ cfu g^−1^) was 0.03% and significantly different compared to 0.26% (0.69 and 0.33% at days 7 and 9, respectively) for spinach F1 Trumpet (*p* = 0.048). The *Carnobacteriaceae* content of the remaining groups ranged from 0.00 to 0.01%. *Listeria* (genus) content was only 0.01% for rocket Esmee and spinach F1 Cello, and not detected in rocket Buzz or spinach F1 Trumpet. When samples with a low number of reads were included in the analysis, *Listeria* was identified in 14 out of all 20 kale Nero di Toscana samples. In stark contrast, *Listeria* was detected in two of 20 samples in rocket Esmee (0.05 and 0.01%), spinach F1 Cello (0.04 and 0.07%), and rocket Buzz (both 0.01%), and in only one of 20 samples belonging to spinach F1 Trumpet (0.02%). Bacillaceae showed a strong negative correlation with *L. monocytogenes* growth in rocket Esmee, but not in Buzz or both spinach varieties. However, differences between Esmee and Buzz were also present at the genus level of Bacillaceae, with *Bacillus* sp. about four-fold higher in Esmee on days 2, 5, and 7 than in Buzz (Supplementary Table S18).

Similarly to findings at 3.1.3, the majority of bacterial phyla and families were detected on spinach, rocket, and kale. Similarly, specific taxa and their change in abundance over time appear to be correlated with *L. monocytogenes* growth.

### 3.3 Comparison 3 (seasonality)

This section describes how alpha and beta diversities were shaped by seasonality (winter and summer) in spinach Trumpet and how diversities evolved during storage. The primary assumption was that winter vs. summer production affects alpha and beta diversities. In turn, differently developing phyllosphere communities were expected to affect *L. monocytogenes* growth over time, as well as the succession of the phyllosphere community over time.

#### 3.3.1 Influence of time of harvest on alpha diversity of a *L. monocytogenes* inoculated spinach phyllosphere

All alpha diversity metrics did not significantly change over time (*p* > 0.05). The number of observed features (ASVs) from summer produce (295–351) and winter produce (308–336) was statistically similar. The same findings were observed for Faith’s Phylogenetic Diversity (18.1–24.9). In contrast, the Shannon index was on average significantly greater for winter produce (6.2–6.8, *L. monocytogenes* growth potential = 1.65 log_10_ cfu g^−1^), compared to summer produce (5.8–6.4, *L. monocytogenes* growth potential = 2.59 log_10_ cfu g^−1^). However, these values did not change significantly over time (days 0–9) for either group (*p* > 0.05). Evenness was also considerably higher for winter produce (0.74–0.81) compared to summer produce (0.72–0.77). Compared to comparisons 1 and 3, here the changes of winter to summer produce had a less pronounced effect on alpha diversity.

#### 3.3.2 Influence of growing season on beta diversity of a *L. monocytogenes* inoculated spinach phyllosphere

**Figure 5:**
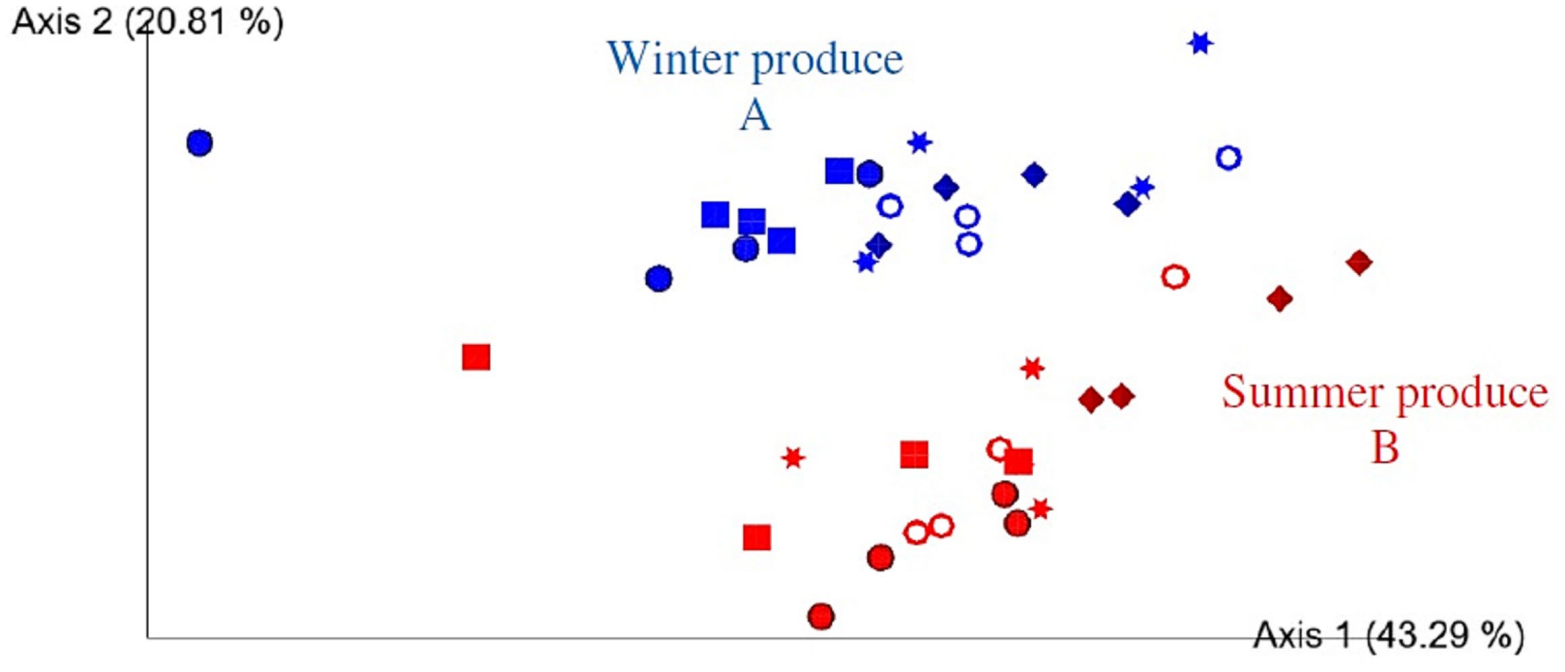
Two-dimensional Emperor (PCoA) plots showing beta diversity distances, that is, weighted UniFrac, among the different samples across open field spinach: winter (blue) and summer (red) produce, with rarefaction applied. Shapes revealed separations over time where day 0 = circle, day 2 = square, day 5 = star, day 7 = ring, and day 9 = diamond. A and B indicate significant differences.

Based on the PCoA plot (Figure 5), separations were visually identified between winter and summer groups over time, evident between all data points, as confirmed by PERMAMOVA (*p* = 0.001). Adjusting the *p*-value significance threshold with Benjamini–Hochberg correction did not alter any significances. Separations for each day 0, 2, 5, 7, and 9 (summer vs. winter) were significant (*p* = 0.026–0.048). A visual separation according to time point within winter and summer produce was also clearly visible (Figure 5). For summer produce, statistically significant separations were observed over time for days 0–9, 2–9, and 5–9 (*p* = 0.022–0.032). For winter produce separations days (0–9, 2–9, 0–7, and 2–7) were significant (*p* = 0.026–0.034). However, after applying Benjamini–Hochberg correction, none of these results remained significantly different. Overall, when compared to alpha diversity, the beta diversity was affected by seasonality.

#### 3.3.3 Influence of time of harvest on phyla and family relative abundance of a *L. monocytogenes* inoculated spinach phyllosphere

For Winter and Summer produce, the most abundant four phyla were *Pseudomonadota*, *Actinobacteriota*, *Bacteroidota*, and *Bacillota*, which comprised 98.94 and 98.86% of the phyllosphere bacterial communities (Supplementary Figure S1). The only significant difference between summer and winter produce at the phylum level was that the *Bacillota* were significantly more abundant in the summer produce (*p* < 0.05). Over time (days 0–9), the total abundance of these four most abundant phyla remained consistent for both groups, ranging from 98.50 to 99.62%.

Winter and summer produce shared 31 families (Supplementary Table S19). However, 20 of those families had significantly different relative abundances between groups (*p* < 0.05). A total of 17 of the most abundant 20 families were shared between both groups, of which eight had significantly different relative abundances, that is, order *Enterobacterales* (family unknown), *Sphingomonadaceae*, *Oxalobacteraceae*, *Rhizobiaceae*, *Caulobacteraceae*, *Nocardioidaceae*, *Rhodanobacteraceae*, and *Nocardiaceae*.

ANCOM revealed 11 differentially abundant families, that is, *Paenibacillaceae*, order *Saccharimonadales* family Unknown, *Myxococcaceae*, *Phormidiaceae*, *Deinococcaceae*, *Rhodobacteraceae*, *Spirosomaceae*, *Moraxellaceae*, *Rhodanobacteraceae*, *Hymenobacteraceae*, and *Nocardioidaceae*, between winter and summer. *Pseudomonadaceae* content was not significantly different between the summer and winter produce (*p* = 0.905) or across all time points from days 0–9 (*p* = 0.075, 0.149, 0.255, 0.051, and 0.527). Similarly, *Lactobacillales* (order level) content was not significantly different between the summer and winter produce (*p* = 0.322) or across days 0–9 (*p* = 0.387, 0.638, 0.773, 0.767, and 0.314). Although *Lactobacillales* relative abundance was less than 1% for all produce, *Lactobacillales* content was on average higher for winter produce (0.34%), compared to summer produce (0.20%). The relative abundance of *Lactobacillales*, that is, *Lactococcus* genus, remained consistent throughout for winter produce, but for summer produce dropped from 0.52 to 0.22 to 0.03% from days 5–9, coinciding with increases in *L. monocytogenes* growth, such levels of *L. monocytogenes* growth which were not observed on winter produce. Moreover, in contrast to polytunnel spinach produce (Comparisons 1 and 2), *Carnobacteriaceae* was not present on open field spinach produce from summer or winter produce (Supplementary Table S19).

*L. monocytogenes* populations for the summer and winter groups showed five common negative correlations with the families *Sphingomonadaceae*, *Microbacteriaceae*, *Beijerinckiaceae*, *Nocardiaceae*, and *Nocardioidaceae.* Seven common positive correlations were identified with families *Pseudomonadaceae*, *Sphingobacteriaceae*, *Weeksellaceae*, an unknown family (*Enterobacterales* order), *Rhizobiaceae*, *Oxalobacteraceae*, and *Xanthomonadaceae* (Supplementary Tables S20, S21). *L. monocytogenes* populations of winter produce had a strong to very strong positive correlation with six families and a strong to very strong negative correlation with five families. *L. monocytogenes* populations in summer produce showed a strong to very strong positive correlation with six families and a strong to very strong negative correlation with four families (Table 5 and Supplementary Tables S20, S21). Similar to findings at 3.1.3 and 3.2.3, the majority of bacterial phyla and families were detected on spinach summer and winter produce. Again, specific taxa and their change in abundance over time appear to be correlated with *L. monocytogenes* growth. Although detected and enumerated on *Listeria* selective agar, the *Listeria* genus, belonging to the *Lactobacillales* order, was not detected using NGS on either winter or summer open field spinach produce (F1 Trumpet variety).

**Table 5:** Average relative abundance (% ± standard errors) of families (16S rDNA) of the summer open field spinach variety F1 Trumpet produce across days 0, 2, 5, 7, and 9 rarefied with strong to very strong Pearson’s correlation coefficient (i.e., the strength and direction of the relationship between that specific family’s relative abundances and the corresponding *Listeria monocytogenes* populations over time). Letters a–d indicate significant differences.

	Day 0	Day 2	Day 5	Day 7	Day 9	Pearson’s correlation
Winter produce						
*Sphingomonadaceae*	13.33 ± 3.57^a^	12.64 ± 0.30^a^	10.87 ± 1.84^a^	11.29 ± 1.73^a^	10.98 ± 1.32^b^	−0.93, very strong
*Pseudomonadaceae*	7.35 ± 2.10^a^	9.86 ± 1.20^ab^	12.03 ± 2.61^ab^	19.28 ± 1.00^b^	19.01 ± 2.05^b^	+0.89, strong
*Microbacteriaceae*	7.77 ± 1.13^a^	8.38 ± 0.62^a^	6.09 ± 1.42^a^	5.47 ± 0.67^a^	5.80 ± 0.56^a^	−0.82, strong
*Beijerinckiaceae*	20.05 ± 5.25^a^	13.57 ± 1.30^a^	6.45 ± 1.09^ab^	6.82 ± 1.51^ab^	7.04 ± 1.13^ab^	−0.93, very strong
*Sphingobacteriaceae*	4.08 ± 1.54^a^	4.90 ± 0.43^a^	9.18 ± 1.48^b^	9.37 ± 0.79^b^	9.10 ± 0.89^b^	+0.92, very strong
*Nocardiaceae*	12.51 ± 1.74^a^	9.58 ± 1.19^ab^	6.73 ± 1.15^b^	6.64 ± 1.23^b^	8.20 ± 0.67^ab^	−0.84, strong
Unknown family	1.56 ± 0.71^a^	4.07 ± 1.06^a^	5.31 ± 1.54^a^	9.03 ± 3.53^a^	6.61 ± 0.47^a^	+0.83, strong
(*Enterobacterales* order)						
*Rhizobiaceae*	3.14 ± 0.11^a^	3.71 ± 0.79^a^	4.49 ± 1.22^a^	3.57 ± 0.32^a^	4.70 ± 0.45^a^	+0.82, strong
*Oxalobacteraceae*	1.06 ± 0.35^a^	1.40 ± 0.16^a^	1.91 ± 0.36^a^	1.93 ± 0.33^a^	1.82 ± 0.37^a^	+0.91, very strong
*Nocardioidaceae*	6.24 ± 3.17^a^	2.93 ± 0.23^ad^	1.71 ± 0.12^acd^	1.60 ± 0.32^bcd^	1.28 ± 0.24^bc^	−0.93, very strong
*Rhodanobacteraceae*	1.57 ± 0.37^a^	2.60 ± 0.39^a^	2.92 ± 0.64^a^	2.77 ± 0.88^a^	2.97 ± 1.07^a^	+0.91, very strong
Summer produce						
*Sphingomonadaceae*	19.71 ± 1.10^a^	17.15 ± 2.12^a^	16.99 ± 2.12^a^	16.45 ± 2.59^a^	12.83 ± 2.03^a^	−0.95, very strong
*Microbacteriaceae*	10.27 ± 1.42^a^	8.01 ± 0.98^a^	7.37 ± 0.41^a^	7.31 ± 1.12^a^	7.25 ± 1.19^a^	−0.71, strong
*Beijerinckiaceae*	7.80 ± 1.02^a^	11.93 ± 3.36^a^	8.93 ± 2.42^a^	6.52 ± 1.30^a^	3.16 ± 0.51^a^	−0.82, strong
*Sphingobacteriaceae*	3.09 ± 1.23^a^	3.62 ± 1.55^a^	5.80 ± 1.75^a^	7.06 ± 1.64^ab^	11.70 ± 1.35^b^	+0.99, very strong
*Weeksellaceae*	2.66 ± 1.00^a^	3.95 ± 1.65^a^	3.95 ± 0.96^a^	3.75 ± 0.76^a^	6.06 ± 0.84^a^	+0.89, strong
Unknown family	1.13 ± 0.38^a^	1.69 ± 0.57^a^	3.00 ± 0.56^a^	5.56 ± 2.70^a^	5.56 ± 1.82^a^	+0.93, very strong
(*Enterobacterales* order)						
*Oxalobacteraceae*	2.59 ± 0.46^a^	2.15 ± 0.61^a^	2.72 ± 0.55^a^	2.70 ± 0.78^a^	4.19 ± 0.71^a^	+0.88, strong
*Xanthomonadaceae*	2.09 ± 0.58^a^	2.64 ± 1.01^a^	2.17 ± 0.58^a^	2.91 ± 0.43^a^	3.21 ± 0.64^a^	+0.84, strong
*Flavobacteriaceae*	0.07 ± 0.01^a^	0.19 ± 0.06^ab^	1.32 ± 0.31^ab^	1.71 ± 1.49^ab^	4.50 ± 2.12^b^	+0.98, very strong
*Hymenobacteraceae*	10.01 ± 1.88^a^	5.61 ± 0.42^a^	4.31 ± 0.64^a^	3.19 ± 0.67^a^	2.09 ± 0.84^a^	−0.86, strong

## 4 Discussion

The purpose of this study was to describe the influence of leafy vegetable cultivation conditions (cultivation method, plant species, cultivar, and season of harvest) on the development of the phyllosphere bacteriome and the effect on epiphytic *L. monocytogenes* growth.

### 4.1 Effects of cultivation conditions (open field vs. polytunnel), plant species (spinach and rocket), and cultivars (varieties)

Previous research assessing the effect of nitrogen fertilizer and leaf mineral content revealed that plant species alone, like spinach and rocket, influence the development of the phyllosphere (Darlison et al., 2019). However, the current study further revealed that the vegetable cultivation method had the strongest influence on the bacterial phyllosphere community structure. At the same time, plant species had a more pronounced effect on the overall abundance of phyllosphere bacteria. Here, polytunnel and open field cultivation of rocket and spinach displayed more similar phyllosphere bacterial communities compared to plant species alone. Additionally, the phyllosphere bacterial communities of various rocket and spinach cultivars were found to be significantly different in the present study. Previous research has identified the presence of microbe-plant variety interactions in field-grown lettuce. Dominated by *Pseudomonadaceae* and *Enterobacteriaceae* families, a clone library of three lettuce cultivars revealed significant differences between the relative abundances of genera belonging to the *Enterobacteriaceae* family, including *Erwinia* and *Enterobacter* (Hunter et al., 2010). While another study of the microbial diversity and structure of the phyllosphere of Alfalfa (*Medicago sativa L.*) identified significant effects of the season and the site where the plants were cultivated in open fields, no significant differences were detected between the two tested varieties (Zhang et al., 2022). While lettuce and alfalfa are bred for cultivation, and both plants are cultivated through a broad range of varieties, only lettuce is bred with the aim of human consumption of the leaves, as is the case for spinach and rocket. One may speculate that the breeding focus of lettuce, spinach, and rocket is primarily on the consumer experience of eating the leaves; thus, different varieties may differ more substantially in their leaf structure than this is the case for other plant varieties that are bred for livestock feeding.

### 4.2 Correlations between *in situ* phyllosphere taxa and inoculated *L. monocytogenes* growth

A novel aspect of the present study was the identification of the presence or absence of bacteria, and their shifts in relative abundance, which may be of potential importance to the *L. monocytogenes* growth. For example, *Pseudomonadaceae*, which are of high abundance and are associated with the hydrolysis of proteins into amino acids, can induce the stimulation of *L. monocytogenes* growth (Marshall et al., 1992; Zilelidou and Skandamis, 2018). Contrariwise, *Lactobacillales* that were present in low abundance are commonly associated with decreased *L. monocytogenes* survival due to their competitive growth abilities (Østergaard et al., 2014). Indeed, the *L. monocytogenes* growth-enhancing *Pseudomonas* species has previously been associated with spinach leaves of neutral pH (Babic et al., 1996). Additionally, as *Pseudomonas* species are pectolytic, their presence is positively correlated with the degradation and spoilage of such leafy vegetables, which increases during storage, as observed in the present study. Exposure to solar active radiation influenced the relative abundance of the *Betaproteobacteria* and *Gammaproteobacteria*, which is the class level of the *Pseudomonadales* order (Truchado et al., 2017). Relative abundances of *Gammaproteobacteria* were not significantly different with reductions in cumulative photosynthetically active radiation (PAR) from 4,889 to 3,602 μmol m^−2^ s^−1^, but were substantially higher when cumulative PAR was 3,115 μmol m^−2^ s^−1^. In the present study, the protection of spinach and rocket produce from PAR by cultivating in a polytunnel setting, compared to an open field, did not lead to significantly higher *Pseudomonadaceae* content.

In the present study, *L. monocytogenes* populations of all groups were positively correlated with *Pseudomonadaceae* content. In particular, *Pseudomonadaceae* content appeared to be most important for *L. monocytogenes* growth on spinach F1 Trumpet produce, especially from day 7 to 9. Relative increases from days 7–9 for open field spinach produce were associated with *L. monocytogenes’* most significant increase during the same period. Conversely, when *Pseudomonadaceae* content decreased from days 7–9 for polytunnel spinach, the *L. monocytogenes* populations remained stationary. Indeed, amino acids hydrolyzed from proteins by *Pseudomonadaceae* are localized within the cellular tissue of leafy vegetables (Koseki and Isobe, 2005; Vacher et al., 2016). Open field spinach produce is likely exposed to more liquids on leaf surfaces due to wetter outdoor climatic conditions, potentially causing higher leaching of those nutrients for *L. monocytogenes* utilization compared to polytunnel produce (Tukey, 1970; Comte et al., 2012; Vacher et al., 2016; Kyere et al., 2019; Zhu et al., 2022). Overall, spinach contained higher total abundances of *Pseudomonadaceae* than rocket. The leaf physiology of rocket, that is, less surface area and fewer stomata (Maylani et al., 2020) might have prevented the release of some nutrients, that is, amino acids (hydrolyzed protein) for *L. monocytogenes* utilization (Culliney and Schmalenberger, 2022), thus limiting the growth of bacteria more than this is the case for spinach. However, at the genus level, higher numbers of *Pseudomonas* sp. on Esmee than on Buzz do not seem to influence the growth potentials of *L. monocytogenes*.

Moreover, a higher *Lactobacillales* content was associated with the lower *L. monocytogenes* growth potential compared to both rocket Buzz and spinach F1 Trumpet produce. A recent study of a mixed spinach salad containing chicken meat identified low levels of *Lactobacillales* content, consisting of only *Carnobacteriaceae* and *Enterococcaceae*, which increased from 0 to 1% at day 7 of storage at 15°C (Söderqvist et al., 2017a). There, the authors did not detect any *Lactobacillales* on plain baby spinach. In another study, storage of romaine lettuce over 14 days revealed a significant increase in *Carnobacteriaceae*’s relative abundance from 1.93 to 52.26% and a non-significant increase in *Pseudomonadaceae* content from 13.38 to 21.20% (Dharmarha et al., 2019). Both bacteriocin-producing, for example, Divercin AS7 and non-bacteriocin-producing species of Carnobacteria, *C. divergens* and *C. maltaromaticum*, have been demonstrated to be effective *in vitro* at minimizing epithelial cell invasion caused by *L. monocytogenes* Scott A (Pilchová et al., 2016) and *Listeria* spp. (Marković et al., 2022). *Carnobacteria piscicola LK5* and *2762* strains suppressed the maximum population density reached by *L. monocytogenes* in brain heart infusion broth (Buchanan and Bagi, 1997). However, little of the *L. monocytogenes* maximum population density suppression was due to the strain’s bacteriocin production. Those authors suggested that the suppression potential of the strain *C. piscicola 2762* was not caused by peroxide, pH depression, or oxygen depletion, but was caused by induced nutrient depletion. In the present study, *Carnobacteriaceae* were absent from open field spinach produce but present in significantly higher quantities on polytunnel spinach produce, particularly at days 7 and 9. This may have also inhibited the growth *L. monocytogenes*, leading to its lower growth potential. Moreover, spinach F1 Cello variety had no *Carnobacteriaceae* present, but significantly higher *Pseudomonadaceae* content (+9.43%) compared to spinach F1 Trumpet from the polytunnel setting. Thus, potentially explaining the higher growth potential of the spinach F1 Cello variety. However, albeit a higher *Pseudomonadaceae* content (+5.58%), polytunnel spinach F1 Cello may have caused less leaching of nutrients (hydrolyzed amino acids) due to being less exposed to rain and liquid on surface of the leaf, thus resulting in lower *L. monocytogenes* growth potential for spinach F1 Cello (1.84 log_10_ cfu g^−1^) compared to open field spinach F1 Trumpet (2.59 log_10_ cfu g^−1^).

### 4.3 Effect of cultivation conditions (open field, polytunnel, species, and variety) and bacterial taxa abundance on *L. monocytogenes* growth potential

In addition to *Carnobacteriaceae*, polytunnel spinach F1 Trumpet, which displayed a lower growth potential of *L. monocytogenes*, showed an increasing trend in *Pectobacteriaceae* content (genus *Dickeya*). In contrast, open field spinach, which was associated with a larger *L. monocytogenes* growth potential, exhibited a decreasing trend in *Pectobacteriaceae* content (genus *Dickeya*). Additionally, polytunnel spinach F1 Cello, which had a decreasing trend of *Pectobacteriaceae* content (genus *Dickeya*), was associated with a higher *L. monocytogenes* growth potential than polytunnel spinach F1 Trumpet. Similarly, rocket Esmee had an increasing trend in *Pectobacteriaceae* content (genus *Dickeya*), whereas rocket Buzz, with a consistently lower *Pectobacteriaceae* content (genus *Dickeya*), was associated with higher *L. monocytogenes* colonization. *Pectobacteriaceae* spp., in particular the genus *Dickeya*, is a necrotroph that is known to cause soft rot, where deterioration of vegetables occurs from the secretion of plant cell wall-degrading enzymes (Bellieny-Rabelo et al., 2019; Wasendorf et al., 2022). Additionally, *Pectobacterium* spp. are associated with a type VI secretion system, which also targets plant pathogens lacking cognate immunity proteins by secreting bactericidal effectors and further releasing low molecular weight bacteriocins, that is, carocin, pectocin, and carotovoricin (Shyntum et al., 2019). Moreover, *Pectobacterium*, *Dickeya*, and *Serratia* spp. produce the β-lactam antibiotic carbapenem (1-carbapen-2-em-3-carboxylic acid). However, leafy vegetable isolates of *Pseudomonas* sp., which putatively influenced *L. monocytogenes* growth on spinach in this study, have been found to possess antibiotic resistance genes toward β-lactam antibiotics such as meropenem and colistin (Yin et al., 2022).

### 4.4 Factors that affect the *L. monocytogenes in situ* growth

In 2016, *Pectobacteriaceae* was added to the *Enterobacterales* order. Prior to this, only a single *Enterobacteraceae* family existed for that order (Adeolu et al., 2016). In the present study, an unknown family from the *Enterobacterales* order was identified, with a relative abundance ranging from 0.00 to 33.46%. While the current study has no particular information on this new taxonomic bacterial group, the *Enterobacteraceae* of the same order possess the ability to produce colicins and microcins (Rebuffat, 2011). Microcins have proven ineffective against *L. monocytogenes*, but colicins produced with the help of the ColE1 gene are highly effective as an anti-listerial agent (Marković et al., 2022). *Enterobacter* spp., particularly *Enterobacter cloacae*, isolated from shredded iceberg lettuce, significantly reduced *L. innocua* colonization due to its nutritional competitiveness (Francis and O’Beirne, 2002).

Darlison et al. (2019) suggested that the influence of phyllosphere diversity on the proliferation of foodborne pathogens such as *L. monocytogenes* should be determined. Indeed, the significantly higher alpha diversity (Shannon index) of produce essentially appears to be correlated with lower *L. monocytogenes* growth potentials in the current study. However, the more diverse polytunnel rocket Buzz variety had more *L. monocytogenes* growth than the rocket Esmee variety. Indeed, higher and increasing *Pectobacteriaceae* content of Esmee, compared to the consistently low *Pectobacteriaceae* content, may be responsible for the 0.22 log_10_ cfu g^−1^ difference between those two growth potentials.

In the current study, seasonality was a significant driver of phyllosphere development in spinach. Bacterial diversity of the phyllosphere of *Typha latifolia* plants was not meaningfully influenced by short-term perturbations in weather conditions, such as rain events, but somewhat affected by seasonal climatic conditions and leaf-associated changes (Stone and Jackson, 2020). Darlison et al. (2019) suggested that annual variations resulting from varying weather conditions influenced phyllosphere communities of rocket and spinach. Although they could not rule out the effect of site-specific factors, as the produce was sampled in different parts of the same field over the 2 years. The present study accounted for site-specific factors by cultivating from the same location within both field and polytunnel settings, and also observed that weather parameters significantly influenced the spinach phyllosphere. Recently, the spinach phyllosphere has also been shown to be substantially influenced by seasonality (PERMANOVA, *p* < 0.003) (Ibekwe et al., 2021). An additional study revealed that the bacterial colonization of lettuce and rocket phyllosphere is also driven, at least in part, by seasonality (Dees et al., 2015).

### 4.5 Effects of total abundances of phyllosphere bacteria across plant species

To date, no previous studies have described the kale phyllosphere. Nevertheless, the kale endosphere has been recently studied (McNees et al., 2020). Across three different brands of store-purchased kale, Illumina sequencing of their endospheres revealed two common dominating Operational Taxonomic Units (OTUs) present were *Pseudomonas* and *Enterobacteriaceae*. In the present study, for kale, these, along with *Micrococcaceae* were also dominating families. Kale Nero di Toscana had the most similar content of *Pseudomonadaceae* as spinach F1 Cello. Although it demonstrated higher *L. monocytogenes* growth, due to the lower TBCs of kale (i.e., 2.80–4.74 log_10_ cfu g^−1^) and lower diversity, compared to rocket and spinach, less inhibition of the *L. monocytogenes* growth potentially occurs due to less competition for resources required for growth. Utilization of chloroplast-excluding protocols at the polymerase chain reaction (PCR) stage COMPETE (RInvT primer) (McManamon et al., 2019) or BLOCK (pPNA clamp) (Fitzpatrick et al., 2018; Culliney and Schmalenberger, 2024) as employed for open field spinach produce in a recent study, and would have been appropriate for rocket Esmee and kale Nero di Toscana. Their chloroplast-to-total DNA content was high, ranging from 58.00 to 97.30% (rocket Esmee) and 92.27 to 99.75% (kale Nero di Toscana), and thus could have prevented the exclusion of the 7 and 16 samples, respectively. Using the NGS approach, *Listeria* content was regularly detected on kale, but rarely occurred for rocket and spinach produce. The high TBCs of spinach and rocket may have contributed to this observation. Moreover, cultivation methods may not have detected cells that were at their viable but not-culturable (VBNC) stage (Müller and Ruppel, 2014). Thus, TBCs for all produce, including kale, may have been underestimated and, therefore, their total DNA content may have been associated with higher actual abundances. For example, a previous study used quantitative PCR (qPCR) and culturable techniques (TSA) to analyze lettuce samples from the same field and revealed that only 0.1–8.4% of TBCs were culturable bacteria (Rastogi et al., 2010). qPCR methods may be used in the future to enumerate TBC for this reason. However, qPCR-based quantifications may potentially overestimate bacterial population densities due to chloroplast co-amplification (Culliney and Schmalenberger, 2022) and multiple 16S ribosomal RNA (rRNA) gene copies per bacterial cell (Schmalenberger et al., 2001); hence, cultivation-dependent and independent approaches have biases. Furthermore, primer selection for 16S rRNA gene-based amplicon sequencing may also be responsible for an additional due to primer mismatch, which appears to be the case for *L. monocytogenes* 16S with the popular V3–V4 primers.

### 4.6 *Pseudomonadaceae* and *Lactobacillales* with putative contradicting effects on *L. monocytogenes* growth

Ibekwe et al. (2021) revealed that the four common dominating phyla *Proteobacteria*, *Firmicutes*, *Bacteroidetes*, and *Actinobacteria*, which comprised 66.35% of their phyllosphere, were significantly different from the overall abundance, ranging from 88.21 to 99.92% of those four main phyla for spinach in the present study. Additionally, their Pseudomonadaceae content (0.49–11.5%) was, on average, lower than the *Pseudomonadaceae* content observed on spinach, rocket, and kale produce in this study. With an overall relative abundance of 35–53%, Pseudomonas has been referred to as the most commonly occurring genus in the spinach and rocket phyllospheres, even after being harvested in different seasons (spring and autumn) (Rosberg et al., 2021). Upon closer inspection of the *Pseudomonadaceae* family’s relative abundances, potential seasonal effects exist, especially for spinach. However, in the current study, the relative abundances of winter and summer open field spinach did not show significant differences. However, there was still a large difference of 0.94 log_10_ cfu g^−1^ between their *L. monocytogenes* growth potentials. LAB are more commonly detected on leafy produce cultivated in spring and summer compared to autumn and winter (Caponigro et al., 2010); however, the opposite was true in the current study. With a relative abundance of less than 1%, *Lactobacillales* may have been responsible for the significant growth potential difference. The *Lactobacillales* decreased from 0.52 to 0.22 to 0.03% from days 5–9 for summer produce, which was correlated with large increments in *L. monocytogenes* growth, which did not occur when *Lactobacillales* remained constant and on average in higher relative abundance for winter produce. More specifically, winter produce with lower *L. monocytogenes* growth had a significantly higher content of the *Lactococcus* genus (*Streptococcaceae* family; *Lactobacillales* order). Indeed, *L. lactis* subsp. *lactis* has been previously isolated from rocket leaves and is known as a bacteriocinogenic strain due to its ability to produce lantibiotic, which is an antimicrobial nisin variant that is highly effective as an anti-listerial agent on food products, including iceberg lettuce (Franz et al., 1997; Kruger et al., 2013; Ho et al., 2018; McManamon et al., 2019; Ho et al., 2021).

The remainder of phyllosphere-associated bacteria, which showed positive and negative correlations with *L. monocytogenes* populations identified in this study, did not appear to be potentially responsible for the conflicting epiphytic *L. monocytogenes* growth on spinach or rocket leaves. Correlations were determined using Pearson’s correlation, which is primarily used for linear relationships between two continuous variables, due to the normal distribution and increasing *L. monocytogenes* populations over time. However, a recent study revealed that Pearson’s can also be more efficient in testing a monotonic nonlinear relation compared to Spearman’s (van den Heuvel and Zhan, 2022). Future studies may use Spearman’s correlation as it evaluates the monotonic relationship between two continuous variables (Schober et al., 2018). Indeed, this approach is most often used for bacterial growth curves, which reach the stationary phase. In either case, such correlations must be interpreted with caution. For example, Zhao et al. (2021) revealed that association means that one variable provides information about another, whereas correlation means that two variables show an increasing or decreasing trend. Therefore, correlation implies an association, but not causation. Additionally, due to the absence of absolute numbers upon sequencing (Gloor et al., 2017), comparing relative abundances could lead to inaccurate conclusions when comparing phyllosphere microbiome over time or when comparing different phyllosphere communities, for example, kale or spinach, which have considerably different absolute cfu data, as relative data reflect a different amount of absolute numbers. Future studies should conduct correlations between absolute cfu data, that is, total bacterial populations and relative abundances from NGS datasets that are converted into absolute values via an additional qPCR step.

## 5 Conclusion

This study identified a link between leafy vegetable species, variety, and environmental growth conditions and the bacterial communities present on the leaf surface, that is, *Pseudomonadaceae*, *Pectobacteriaceae*, and *Lactobacillales*, such as *Streptococcaceae* and *Carnobacteriaceae*. Together, these factors are important in determining the growth potential of *L. monocytogenes*. However, the *Pseudomonadaceae* content appeared to be less critical for plant species with specific leaf surface characteristics, such as a narrow leaf surface area and a smaller number of stomata (e.g., rocket). Therefore, future studies should include leaf surface analyses in growth studies of *L. monocytogenes* on leafy vegetables. Due to the limitations of second-generation sequencing technologies in determining species-level identification of bacteria, a sequencing approach using third-generation amplicon sequencing techniques, as well as true metagenomics approaches, may reveal further insights into the functions of certain bacterial taxa in the phyllosphere and their abilities to aid or retard the *L. monocytogenes* growth.

Advancing aspects of microbial food safety for leafy vegetables may include future selection of varieties that are not only preferred due to their taste and sensory input during consumption but also due to their beneficial natural microbiome. Similarly, one could imagine a future where leafy vegetables are treated with probiotic foliar applications, where beneficial microbes are designed not only to be helpful for digestion but also helpful in suppressing foodborne pathogens.

EURL’s guidance document requires three batches for assessment of the growth potential of RTE products. These three batches are recommended to be from different production days. Although based on results from this study, this should be further updated to reflect produce with different seasonality. Moreover, as identified in the present study for spinach and rocket, the presence of certain phyllosphere or microbiome members could provide more in-depth information regarding *L. monocytogenes* growth potentials on RTE food products than TBC. Thus, the inclusion of NGS techniques could be considered an essential tool for assessing future challenges.

Microbiologists looking to describe the phyllosphere of kale or rocket (Esmee variety) should consider the use of chloroplast amplification blocking methods. This will reduce the number of samples discarded due to low bacterial reads, as occurred in the present study, thereby providing more detailed descriptions of phyllosphere-associated bacteria.

## Data Availability

The datasets presented in this study can be found in online repositories. The names of the repository/repositories and accession number(s) can be found at: https://www.ncbi.nlm.nih.gov/genbank/, PRJNA1177234.
